# Destabilized 3’UTR elements therapeutically degrade ERBB2 mRNA in drug-resistant ERBB2+ cancer models

**DOI:** 10.3389/fgene.2023.1184600

**Published:** 2023-06-09

**Authors:** Chidiebere U. Awah, Yana Glemaud, Fayola Levine, Kiseok Yang, Afrin Ansary, Fu Dong, Leonard Ash, Junfei Zhang, Olorunseun O. Ogunwobi

**Affiliations:** ^1^Department of Biological Sciences, Hunter College of The City University of New York, New York City, NY, United States; ^2^Joan and Sanford I. Weill Department of Medicine, Weill Cornell Medicine, Cornell University, New York, NY, United States; ^3^Department of Pathology and Cell Biology, Department of System Biology, Columbia University Medical Center, New York, NY, United States

**Keywords:** ERBB2 (HER2/neu), 3'UTR elements, cancer, drug resistance, cancer treatment, mRNA stabilizing, mRNA overwriting

## Abstract

Breast, lung, and colorectal cancer resistance to molecular targeted therapy is a major challenge that unfavorably impacts clinical outcomes leading to hundreds of thousands of deaths annually. In ERBB2+ cancers regardless of the tissue of origin, many ERBB2+ cancers are resistant to ERBB2-targeted therapy. We discovered that ERBB2+ cancer cells are enriched with poly U sequences on their 3’UTR which are mRNA-stabilizing sequences. We developed a novel technology, in which we engineered these ERBB2 mRNA-stabilizing sequences to unstable forms that successfully overwrote and outcompeted the endogenous ERBB2 mRNA-encoded message and degraded ERBB2 transcripts which led to the loss of the protein across multiple cancer cell types both in the wildtype and drug-resistance settings *in vitro* and *in vivo*, offering a unique safe novel modality to control ERBB2 mRNA and other pervasive oncogenic signals where current targeted therapies fail.

## Graphical Abstract

**Figure:**
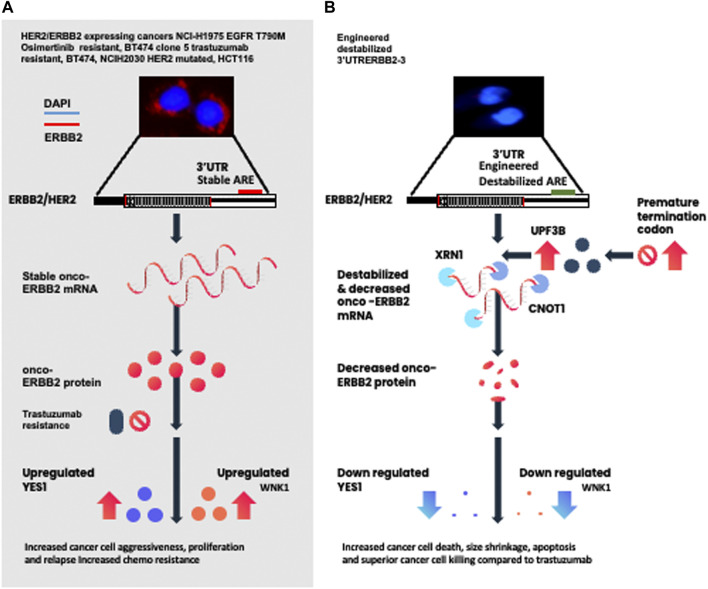
**(A)** Depiction represents multiple ERBB2-expressing cancer cells with stable 3’UTR and the signaling cascade known to cause treatment resistance. **(B)** Depiction of the engineered destabilized 3’UTR of ERBB2 leading to destabilization and the decrease of the ERBB2 transcript, protein, and kinases involved in the mediation of drug resistance.

## Introduction

Breast and lung cancers are deadly tumors afflicting millions of patients worldwide annually. Breast cancer is the most common form of cancer in women worldwide with mortality of approximately 58% in developing countries (WHO, 2022). According to NCI SEER statistics, 53.1 per 100,000 men and women will develop lung and bronchial cancer per year, with a 5-year survival rate of 21.7%. Lung cancer caused more deaths in 2017 than breast cancer, prostate cancer, and brain cancers combined (Siegel et al, 2020). In the United States, the incidence of breast cancer is at 1.6% annual growth rate (AGR) at 267,866 cases per year. In Western Europe (Germany, France, and United Kingdom), the incidence of breast cancer is between 0.39% and 0.98% averaging approximately 50,000–77,000 cases. In China, breast cancer is at 3.36% with 244,949 cases per year, while incidence rates in Japan at 90,452 cases per year. In Africa, breast cancer is at 28% incidence with very poor outcomes and high mortality accounting for 20% of all cancer deaths in women (Sung et al 2021). There is a disparity in breast cancer survival amongst women of African descent when compared to women of other ancestry. A recent study shows that there is a significant disparity across all survival outcome indices in women of African ancestry with ERBB2+ breast cancer in comparison to women of other ancestry with ERBB2+ breast cancer (Zhao et al, 2020).The prevalence of breast and lung cancer and their related mortality arises from the oncogenes that drive the malignant breast and lung tissues to excessively proliferate and then metastasize to lymph nodes and onward to organs such as lungs, liver, and brain leading to the death of the patient.

ERBB2/HER2 is a member of the subclass I receptor tyrosine kinase superfamily of ERBB/EGFR (epidermal growth factor receptor family) which consist of four members, namely, EGFR/ERBB1, ERBB2, ERBB3, and ERBB4 (Salmon et al., 1987; Hynes and Lane, 2005). ERBB2 is activated upon binding of the neuregulin ligand onto the ERBB receptor which leads to its homo and heterodimerization triggering the activation of tyrosine kinases which have docking sites on the ERBB receptors and from there control large-scale signaling proteins, transcription factors, and kinases which mediates ERBB functions. ERBB2/ERBB2 is overexpressed in many human cancers including breast, lung, and colorectal cancers. The overexpression of ERBB2 is associated with very aggressive breast and drug-resistant lung cancer because ERBB2 is a membrane protein that signals and amplifies for proliferation, pro-survival, and prometastatic signals of the cancer leading to poor clinical outcomes.

Tyrosine kinase inhibitors such as osimertinib, erlotinib, and gefitinib target the ERRB/EGFR, and antibodies such as trastuzumab, which target the ecto-domain of ERBB2, have been developed as therapeutics such as ERBB2-overexpressing cancers. Trastuzumab in various forms in combination with chemotherapy has been successful in targeting ERBB2 overexpression in ERBB2+ breast cancer and improving survival as a standard first-line therapy for more than a decade (Molina et al, 2005; Cobleigh et al,1999). However, there are groups of ERBB2+ breast cancer, lung cancer, esophageal cancers, and gastrointestinal cancers that are resistant to trastuzumab and tyrosine kinase inhibitors (Derakhshani et al, 2020; Bose and Ma, 2021; Hanker et al 2021; Zhang, 2021; Li et al, 2022). It is well known that 25% of early-stage and 75% of late-stage ERBB2+ breast cancer is resistant to trastuzumab. The same is true for EGFR T790M non-small-cell lung cancer that is osimertinib-resistant with 6% of patients bearing ERBB2 amplification (Safran et al, 2022; Takeda and Nakagawa, 2019; Vallillo et al., 2020; Soria et al,2018; Sun and Osimertinib, 2018; Monica et al 2017; Zhu et al 2020; Tang, 2018).

For these patients who have treatment-resistant cancers with an overexpression of ERBB2, there is limited clinical intervention for them. More so, for these trastuzumab-resistant ERBB2-driven cancer patients, regional and distant organ metastasis of the tumor is a significant obstacle to good clinical outcomes. Resistance to trastuzumab and other tyrosine kinase inhibitors in ERBB2+ breast and lung cancer is a grand challenge that needs to be overcome to improve clinical outcomes and to prevent drug resistance and help sufferers of these deadly cancers.

To address this grand challenge of ERBB2+ drug resistance in breast and lung cancer, we have developed an innovative approach based on genome engineering of the genetic codes on the 3’UTR (3’ untranslated region) of the ERBB2 gene to destabilize, overwrite/outcompete, and degrade ERBB2 transcript, protein expression, ERBB2-dependent kinases, and interactome. The structure of a typical gene consists of various parts, namely, 5’UTR (untranslated region), the CDS (coding sequence), and the end of the gene called 3’UTR. The 3’UTR of genes serves as a hub for translation, stabilizing, and destabilizing of transcript and polyadenylation (Vejnar et al 2019). Various motifs have been discovered in the 3’UTR region of genes to determine which transcripts are stabilized or destabilized and degraded (Geisberg et al, 2014; Geissler et al 2016). The AU-rich elements (AREs) found on the 3’UTR have been demonstrated to be involved in the nonsense-mediated decay of transcript via a complex process that involves decapping enzymes, deadenylation proteins, and cleaving enzymes (Lykke-Andersen and Wagner 2005).

Nonsense-mediated decay is an eukaryotic universally conserved mRNA surveillance mechanism that targets mRNA transcripts with premature termination codons for targeted degradation. This process is used for mRNA quality control to remove the toxic transcript and for lengthening or shortening the transcript to create mRNA isoforms (Schoenberg and Maquat, 2012; Lykke Andersen and Wagner, 2015). This is a tightly regulated process orchestrated by four distinct steps, namely, mRNA decapping, deadenylation, 5’–3’ and 3’–5’ exonuclease activity, and endonuclease activity. The UPF1–UPF2–UPF3 complex is the master regulator of the nonsense-mediated decay. Proteins such as the human DCP1A, DCP2 and ZFP36, XRN1 (5’–3 exonuclease), and CNOT1 (3’–5’ exonuclease) are involved in this process of ARE-mediated decay (Lykke-Andersen and Wagner 2005). Motifs on the 3’UTR of the gene were found to control mRNA transcript stabilization or destabilization (Lykke-Andersen and Wagner 2005). The consensus stabilization motifs are UUUUU, UUGCAUGG, and CCUUACAC, whereas the destabilization motifs are AUUUU, CCUC, CUGC, UAAGUUAU, UAACUUAU, and GUAAAUAG.

Based on these findings, we hypothesized that the 3’UTR of oncogenes such as ERBB2 will be enriched with poly U mRNA-stabilizing elements, which stabilize their transcripts and drive tumor aggressiveness. Therefore, if we change these stabilizing elements to destabilizing elements by motif engineering and drive them by mRNA decapping promoter DCP1A, we hypothesized that we can control the oncogene by overwriting/outcompeting the endogenous ERBB2 mRNA and degrade the transcript, which will lead to the loss of proteins and signaling cascade. Here, we report data that demonstrate that by destabilizing the stable 3’UTR elements of ERBB2 across multiple recalcitrant drug-resistant cancer models, we controlled pervasive ERBB2 oncogenic transcript and specifically degraded the ERBB2 protein and its associated kinases and interactome which triggered apoptosis and killed the tumors and is validated *in vivo*. These findings uncover a novel technology to target and control oncogenes where drugs failed and for therapeutic gene targeting in diseases and control of any transcript of interest.

## Materials and methods

### Cell culture of BT474, BT474 clone 5, NCI-H1975, NCI H2030, HCT116, and MCF10A

BT474 was grown in DMEM supplemented with 10% FBS and 1% penicillin–streptomycin. The BT474 clone 5 (trastuzumab-resistant) and NCI-H1975 EGFR T790M and NCI H2030 were grown in RPMI-1640 supplemented with 10% FBS and 1% penicillin–streptomycin. HCT116 were grown in McCoy’s 5A modified medium with 10% FBS and 1% penicillin–streptomycin. MCF10A was grown in MEBM with additives as listed in ATCC. The cells were grown to 80% confluency before use.

### RNA extraction from breast cancers

We extracted total RNA from the BT474 using the RNA Easy kit from the Qiagen (cat no: 74104). The RNA was stored at −80°C until use.

### QPCR with ERBB2 3’UTR primers

To determine the mRNA-stabilizing poly U-rich elements on the 3’UTR of ERBB2, we made cDNA from the total RNA of BT474, MCF7, T47D, and MDA MB231 using the Qiagen Reverse Transcription kit (Catalog no: 205311). We performed RT-PCR using the cDNA according to the Qiagen manufacturer’s protocol, and the primers used are in Supplementary Table S1.

### Sanger sequencing of 3’UTR of ERBB2 cDNA amplicon

To confirm the ERBB2-stabilizing 3’UTR poly U sequences, the amplified bands were excised under UV light and then extracted with the Qiagen Gel Extraction kit (Catalog no. 28706X4) according to manufacturer’s protocol. The amplicon was then sequenced with the ERBB2 3’UTR PCR primers using the Sanger Sequencing method by the commercial company Psomagen Inc., Brooklyn, New York, United States (Supplementary Figure S3). Subsequently, we used the online software DNA to the mRNA translator (http://biomodel.uah.es/en/lab/cybertory/analysis/trans.htm) to identify the stable ARE motifs of ERBB2 3’UTR (Supplementary Figure S3). The 3’UTR cDNA sequencing data were deposited (10.5281/zenodo.6968947).

### Design of stable destabilized 3’UTR of ERBB2

To design the destabilized 3’UTR of ERBB2, we replaced the consensus-stabilized UUUUU, UUGCAUGG, and CCUUACAC motifs in the mRNA with destabilized ARE consensus motifs of CCUC, CUGC, and UAAGUUAU, respectively (Supplementary Figure S4). Next, we modified some residues on the 3’ end of the 3’UTR to increase stability. Torabi et al. (2021) used structural biology and biochemical assays to delineate residues of helices of the nucleic acids on the poly A tail of 3’UTR mRNA that determines their strong, moderate stability as well as rapid degradation. With this information, we analyzed the loop structures of the stabilized and destabilized 3’UTR of ERBB2 (Reuter and Matthews, 2010) (Supplementary Figure S5). The stabilized ERBB2 structures have stability determining U-A on the lower loop stem as well as on the lower URIL. We used the mutated residues from Torabi et al (2021) that confer moderate-degree stability as an example. In designing the destabilizing ERBB2 3’UTR, we changed M5 (C–U), M6 (U–C), and M11 (A–U), and all residues are marked in red asterisks. The changes in these residues have a remarkable impact on the stability of the transcript for up to 120 mins (Torabi et al, 2021). Having established the structure of stabilized ERBB2 3’UTR and mutated residue of destabilized ERBB2 3’UTR that increased their stability, we incorporated these destabilized constructs with an upstream 5’ BstB1 restriction site followed by a poly A sequence to stop the RFP transcription of the vector followed by a DCP1A promoter. At the 3’ end, we added a poly A sequence followed by the BamH1 restriction site (Supplementary Figure S6).

### Synthesis of destabilized 3’UTR of ERBB2 as gBlocks

We synthesized the destabilized 3’UTR of ERBB2 as gBlocks from Integrated DNA Technologies (IDT Inc., United States).

### Cloning of destabilized 3’UTR of ERBB2 into the Sp6 vector both by sticky end cloning and Gibson assembly is described in detail in the supplementary methods

We clone the destabilized 3’UTR ARE of ERBB2 into pLenti-CMVSP6-nEGFP-SV40-PURO (Addgene: # 138364).

### Transfection/electroporation of destabilized 3’UTR of ERBB2 into BT474 wildtype, BT474 clone 5 (trastuzumab resistance), and NCI-H1975, NCI-H2030, and HCT116 cancer cells

To introduce the destabilized 3’UTR into the cancer cells, we electroporated constructs into 200,000–500,000 cells with 50 ng of plasmid containing the constructs using the BioRad electroporation system. We used the preset mammalian protocol set for 293 T cells and pulsed the cells in a cuvette 2x. The cells were then seeded into six-well plates and viewed for morphology and red fluorescent protein (RFP) expression in 24 hrs. After 24 hrs, the cells expressed RFP indicating that the constructs were successfully integrated into the cells. The constructs were also injected intraperitoneally to the animals as a plasmid.

### YES1 overexpression

The YES1 Y537F overexpression vector (Addgene: 51299) was transfected into desARE3’UTR ERBB2-3 and -30 cells that have lost ERBB2 and YES1 by introduction of destabilized desARE3’UTR ERBB2. Overexpression of YES1 was confirmed by the YES1 Western blot.

### Cell microscopy

We observed the cells regularly under the light microscope at 20x. Within day 4, the BT474 wildtype containing the destabilized elements showed a distorted and ruptured membrane compared to the control. For the trastuzumab-resistant cells, the changes in cellular morphology were observed from the starting of day 9 for the cells containing destabilized constructs. The cell size was drastically reduced compared to controls.

### Immunofluorescence

To determine the ERBB2, caspases 3 and 9 and cleaved caspase 3 protein expression changes in the destabilized breast and lung cancer compared to the wildtype. We seeded the cells on a tissue culture slide or six-well plate at 2,000–3,000 per well. The cells were allowed to grow for 1 day, and after they were fixed with 200 µl of 4% paraformaldehyde, they were added to the cell media. The cells were placed at 4°C for 5 mins, after which the paraformaldehyde was decanted. Anti-ERBB2 (Human ErbB2/Her2 Mab, Clone 191924 R&D systems) 1:1000 in BSA was added to the slide wells and sealed with an aluminum foil and kept at 4°C overnight. After that, the antibody is removed and secondary antibodies Alexa 488 (green) or Alexa 610 (red) were added at 1:10,000 and covered with the aluminum foil and kept at room temperature for 1h. After 1h, the secondary dye was washed with PBS (phosphate-buffered saline) 3x, and then, the water from edges were wiped off with Kim wipes. In addition, a drop of DAPI (NucBlue nuclear stain) was added and then sealed with cover slips, and the slides were viewed under a Nikon confocal microscope. For the tissue slides, slides returned from the expert pathologists were washed in 1x PBS by dipping and, subsequently, dripped off and cleaned with Kim wipes. Anti-ERBB2, CNOT1, XRN1, and UPF3B (1:1000) were dropped on the slides and covered overnight at 4°C to avoid desiccation. The slides were washed in 1x PBS and Alexa 610 was added and incubated at room temp for 1h. After that, the slides were washed and a drop of DAPI was added and covered with a coverslip, and finally, the image was acquired under a Nikon confocal microscope.

### Western blot

To quantify the ERBB2, YES1, WNK1, and CNOT1 protein expression changes in wildtype cells compared to the cells containing the destabilized 3’UTR ARE. We harvested the cells and lysed them in a cocktail of the protease inhibitor in an MPER buffer. The protein extract was stored in −80°C until use. The protein was separated in 12% stacked SDS page gels and separated by initial run at 75 V and after 15 mins at 120 V until complete separation. The proteins were transferred onto a PVDF membrane already charged with methanol at 25 V for 1 h. The membrane was blocked with BSA and subsequently incubated with anti-ERBB2(Human ErbB2/Her2 MAb Clone 191924) or anti-YES1 and WNK1 (Cell Signaling #65890 and #4979), CNOT1 (14276-1-AP) overnight in BSA. The primary antibody was removed, and secondary anti-mouse IR 800CW dye (Li-COR) was added at 1:1000 incubated with BSA covered from light for 2 hrs. After incubation, the membrane was washed with TBST 3x, and the image was taken on an Li-COR Oddysey chemiluminescent imager.

### Cell viability

To quantify if the destabilized constructs affect the cell survival, we performed cell viability comparing the wildtype cells with the cells carrying destabilized constructs with CellTiter-Glo (Promega cat: G7570).

### Caspase 3/7 assay, caspase 3, cleaved caspase 3, and caspase 9 protein analysis

To assay for increased caspase activity, we used the caspase 3/7 Glo kit (Promega cat: G8090) as well anti-caspases 3 and 9 (Abcam #4051, #25758) and cleaved caspase 3 (Cell Signaling #9664). The kit was used according to the manufacturer’s instructions. We performed immunofluorescence with antibodies against cleaved caspase 3 and caspase 9 on wildtype cells and the engineered destabilized cells as described previously.

### Phosphorylation kinase array

To determine how kinases are affected by the destabilization of the ERBB2 protein, we used the Human Phospho-Kinase Array kit (Cat no. ARY003C). We followed the protocol as described.

### Migration assay

Wounds were made on the cell monolayer using a sterile 200 μL pipette tip. The cells were washed with 1 × PBS, and fresh media were added to each well following the wound. The images of scratched areas were taken at 10× magnification using an AE30 inverted microscope (Motic, Richmond, BC, Canada).

### RNA seq

To confirm the significant downregulation of ERBB2 on the genome scale following the destabilization of the 3’UTR ARE as well as to ascertain to what level the ERBB2 interactome is affected by the significant downregulation of ERBB2, its specificity is compared to the wildtype controls. We performed RNA Seq on the RNA from desARE3’UTR ERBB2-3 and desARE3’UTR ERBB2-30 compared to the wildtype and vector controls. RNA Seq analysis was performed using established pipelines and BioJupies (https://maayanlab.cloud/biojupies/). Data deposited: 10.5281/zenodo.6968947.

### Quantitative reverse transcript PCR

To quantify the ERBB2 expression changes upon destabilization of the ERBB2 3’UTR comparing the wildtype and the vector controls, we performed quantitative PCR on ERBB2, deadenylases CNOT1, XRN1, and PARN, and decapping enzyme DCP1A using their exon primers and GAPDH as control. All primers used are listed in Supplementary Table S1 **.**

### Optical genome mapping

To understand if our engineering method introduced copy number variation, indel, etc, we used optical genome mapping provided by Bionano. (https://bionanogenomics.com/products/saphyr/). The experiments were performed in two independent replicates. Data deposited: 10.5281/zenodo.6968947.

### Animal study

We obtained IACUC approval for the animal study from the institutional board. A total of 25 female NSG mice were purchased from the Jackson laboratory. The animals were received and allowed to acclimate according to the institutional protocol. NCI-H1975 was expanded as described previously, and on the day of implantation, the cells were harvested, washed, and resuspended in phosphate-buffered saline (PBS). The confluency of the cells was 80%. We implanted 5 million cells in PBS on the flank of each mouse. After 35 days, huge tumors engrafted, and on 36 days, we randomized the mice into five mice per cage into five groups and ensured equal distribution of tumor size within each group. The vector and desARE3’UTR ERBB2-1, -3, and -30 were administered as plasmids at 20µg/0.1m per mice intraperitoneally 12 hrly for 9 days with a 1-day dosing break. The group of wildtype NCI-H1975 mice received no treatment. Daily measurements of tumor size (length and width), weight, and body condition score were obtained. At day 46, due to the enormous size of the wildtype and vector tumor sizes, the control groups were euthanized and, subsequently, the mice receiving the constructs were also euthanized to perform complete necropsy, full blood count, and blood chemistry analysis. The necropsy and complete blood count and histopathology were performed by an expert pathologist from the Memorial Sloan Kettering Hospital, New York. They were blinded to the experimental details.

### Full blood count and electrolyte analysis

The full blood count and necropsy and electrolyte analysis were performed by the expert pathologist at the Memorial Sloan Kettering Cancer Center, New York City. See Supplementary Figures S4, S5.

### Statistical analysis

All experiments were performed in multiple replicates. The cell-based experiments were performed more than 3x at the minimum as indicated on the legends, for those n = 2 that show the best results from more than 3x experiments. A total of 25 female NSG mice were used at five mice per group per cage. The groups were WT, vector, and desARE3’UTR ERBB2-1, -3, and -30. All animal data presented were performed as five mice in a cage per group. We used a two-tailed T-test to determine the statistical significance difference between the different treatment groups.

### ImageJ

We used the software ImageJ to quantify the immunofluorescence signal of ERBB2, cleaved caspase 3, and caspase 9.

### GraphPad prism

We used the GraphPad Prism software to draw all the bar charts presented and performed all statistics with the software.

### 4SU mRNA labeling pulse-chase experiment

To understand how the destabilized exogenous constructs destabilize the endogenous ERBB2 mRNA, we seeded in a six-well plate 1 million cells of the NCI-H1975 WT, NCI-H1975 vector, and desARE3’UTR ERBB2-3 and -30 and treated them with 4SU at 1ug/ml and collected cells at 0 h, 30 mins, 1 h, 3 h, 6 h, and 24 h. We collected RNA, made cDNA, and performed RT-qPCR with primers targeting the endogenous ERBB2 as well as primers targeting the vector as well as RFP and the cloned destabilized ERBB2, and the control primer is ACTB. We calculated delta Ct (Ct target-Ct destabilized ERBB2) and fold enrichment as 0.5^Ct. We found that the destabilized ERBB2 transcript is 2.5 × 10^4^ overexpressed than the endogenous ERBB2.

## Results

### Identification of mRNA poly U stabilizing motifs and design of the engineered destabilized 3’UTR ERBB2 constructs

**FIGURE 1:**
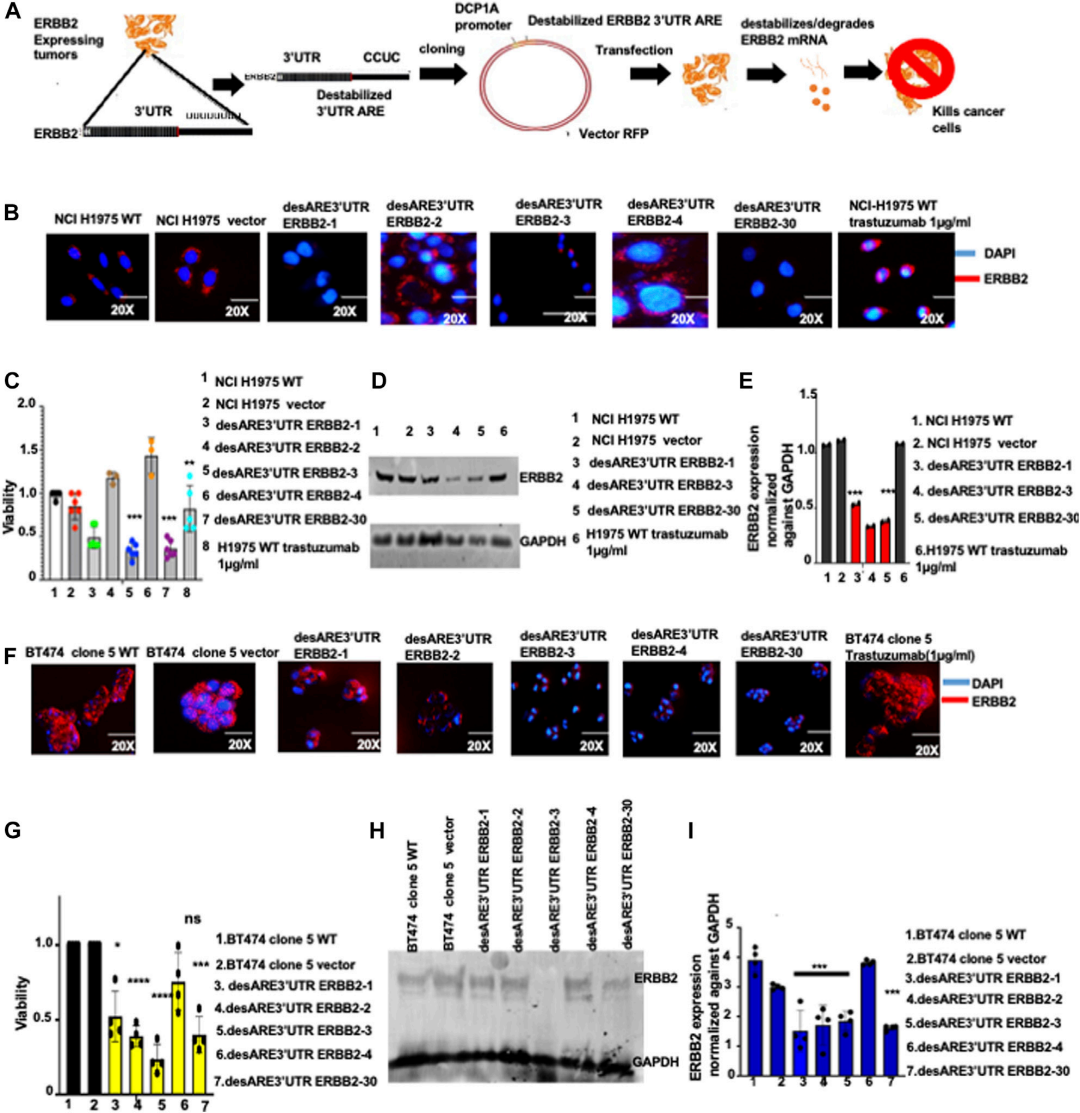
Destabilized 3’UTR of ERBB2 degrades the oncogenic ERBB2 protein and transcript in EGFR T90M-mutated lung cancer and in ERBB2+ trastuzumab-resistant breast cancer leading to cell death. **(A)** Schematic depiction of the destabilization technology, ERBB2-expressing cancers. RNA is extracted and 3’UTR cDNA is made and sequenced by the Sanger method, and the poly U sequences are identified. The poly U sequences on the 3’UTR are engineered and changed to the destabilized motif which is cloned into an RFP vector and driven by DCP1A. The constructs are transfected into ERBB2-expressing cells, and mRNA transcript destabilization is established within 4 days, the cellular membranes denude, and the cancer cell is killed within 8 days. **(B)** Immunofluorescence pictures show ERBB2 expression (stained in red) and nuclei (DAPI-blue) on wildtype NCI-H1975, vector, and on NCI-H1975 containing constructs desARE3’UTR ERBB2-1, -2, -3, -4, and -30 and wildtype-treated trastuzumab NCI-H1975 (n = 2). Magnification is 20x. **(C)** Bar charts show cell viability of the NCI-H1975 wildtype cells, vector, and cells containing constructs desARE3’UTR ERBB2-1, -2, -3, -4, and -30 and wildtype trastuzumab-treated cells (n = 2). Two-tailed T-test (***p-value = 0.0013 WT, vector vs. desARE3’UTR ERBB2-3, **p-value = 0.03 WT, vector vs. desARE3’UTR ERBB2-30). **(D)** Western blot show ERBB2 and GAPDH expression across the NCI-H1975 wildtype cells, vector, cells containing constructs desARE3’UTR ERBB2-1, -3, and -30 and wildtype trastuzumab-treated NCI-H1975 cells (n = 2). **(E)** Bar charts show quantification of the ERBB2 expression normalized against GAPDH by qPCR on NCI-H1975 wildtype cells, vector and cells containing constructs desARE3’UTR ERBB2-1, -2, -3, -4, and -30 and wildtype trastuzumab-treated NCI-H1975 cells (n = 2). Two-tailed T-test (***p-value = 0.007569 WT, vector vs. desARE3’UTR ERBB2-3, ***p-value = 0.001123 WT, vector vs. desARE3’UTR ERBB2-30). **(F)** Immunofluorescence images show ERBB2 expression (stained in red) and nuclei (DAPI-blue) on wildtype BT474 clone 5, vector, and on BT474 clone 5 containing constructs desARE3’UTR ERBB2-1, -2, -3, -4, and -30 and BT474 clone 5 WT trastuzumab treated (n = 2). Magnification is 20x. **(G)** Bar charts show cell viability of the BT474 clone 5 wildtype cells, vector, and cells containing constructs desARE3’UTR ERBB2-1, -2, -3, -4, and -30 (n = 2). Two-tailed T-test (**p-value = 0.011034 WT, vector vs. desARE3’UTR ERBB2-1, ****p-value = 0.000427 WT, vector vs. desARE3’UTR ERBB2-2, ****p-value = 0.000579 WT, Vector vs. desARE3’UTR ERBB2-3, ns = 0.084504 WT, vector vs. desARE3’UTR ERBB2-4, **p-value = 0.002188 WT, Vector vs. desARE3’UTR ERBB2-30). **(H)** Western blot shows ERBB2 and GAPDH expression across the wildtype BT474 clone 5 WT, vector, and on BT474 clone 5 containing constructs desARE3’UTR ERBB2-1, -2, -3, and -4 (n = 3). **(I)** Bar charts show quantification of the ERBB2 expression normalized against GAPDH by qPCR on BT474 clone 5 wildtype cells, vector, and BT474 clone 5 cells containing constructs desARE3’UTR ERBB2-1, 2, 3, 4 and 30 (n = 2). Two-tailed T-test (***p-value = 0.003, WT, vector vs. desARE3’UTR ERBB2-1, 2, 3 and 30).

We hypothesized that the oncogenic transcript of ERBB2 is stabilized on the 3’UTR with poly U sequence and that if the stabilizing element is destabilized, we could overwrite/outcompete the endogenous ERBB2-encoded message, degrade the ERBB2 transcript and protein expression, and thus control the aggressiveness of ERBB2+-driven cancers both in wildtype and drug-resistant settings. For this purpose, (Figure 1A **)**, we extracted RNA from ERBB2+ and non-ERBB2+ breast cancer cells (BT474, MCF7, MDA MB231, and T47D), performed cDNA synthesis, performed PCR amplification of the cDNA, and then sequenced them with ERBB2 3’UTR primers by Sanger sequencing (Supplementary Figure S3A–E). We found that the 3’UTR of ERBB2 in only ERBB2-expressing cancer cells MCF7, BT474, and T47D (Supplementary Figures S3A, B, C, and E) is enriched with UUUUUU sequences which are mRNA-stabilizing elements (Figure 1A; Supplementary Figures S3A, B, C, E; Supplementary Figures S4A). However, these stabilizing elements are not enriched in triple-negative breast cancer MDA MB231 (Supplementary Figure S3D). We designed a synthetic gBlock of this 3’UTR region, wherein we engineered the various stabilizing elements into destabilized elements and cloned them into a vector (Figure 1A; Supplementary Figure S4A; Supplementary Figure S5A, B; Supplementary Figure S6A, B). To design the destabilized 3’UTR, we replaced all the stable elements with the destabilized ARE elements which are CCUC, CUGC, and UAAGUUAU (Supplementary Figure S4A). We reasoned that if we destabilized the ERBB2-3’UTR and drive its expression by the mRNA decapping enzyme promoter DCP1A, we could overwrite the endogenous ERBB2 mRNA and degrade the transcript, specifically through nonsense-mediated decay, resulting in loss of protein expression. Therefore, we designed the destabilizing ERBB2 synthetic gBlock to contain the BstB1 site, a poly A sequence to stop RFP transcription, and then a DCP1A promoter element to drive the destabilized ERBB2 3’UTR and then on the 3’ end with the Bamh1 site and a poly A to stop DCP1A (Supplementary Figure S6A, B)**.**

### Engineered destabilized 3’UTR of ERBB2 degrades ERBB2 in ERBB2-expressing EGFR T790M lung cancer cells, ERBB2+ trastuzumab-resistant breast cancer cells, in wildtype ERBB2+ breast cancer cells, in ERBB2-mutated lung cancer cells, and in ERBB2-expressing colorectal cancer cells

Our cloning strategy yielded four destabilizing 3’UTR ERBB2 clones by sticky end cloning in recombinant-competent *E. coli*, namely, desARE3’UTR ERBB2-1, desARE3’UTR ERBB2-2, desARE3’UTR ERBB2-3, and desARE3’UTR ERBB2-4 (Supplementary Figure S7A–D) and a clone by Gibson assembly in recombinant-deficient *E. coli*: desARE3’UTR ERBB2-30 (Supplementary Figure S8A). We transfected the four destabilized (des) clones (desARE3’UTR ERBB2-1, desARE3’UTR ERBB2-2, desARE3’UTR ERBB2-3, desARE3’UTR ERBB2-4) and desARE3’UTR ERBB2-30 into non-small-cell lung cancer cells that carry mutation in the EGFR T790M but expresses ERBB2. Within 4 days (Figure 1B), by immunofluorescence staining for ERBB2 (ERBB2 stained in red, and nuclei stained blue with DAPI) and Western blotting detection of ERBB2 (Figure 1D), we found that ERBB2 protein expression was lost in desARE3’UTR ERBB2-1, -3, and -30 compared to the wildtype cells, vector control, and trastuzumab-treated NCI-H1975 cells. The NCI-H1975 cells transfected with desARE3’UTR ERBB2-1, -3, and -30 show significant loss of viability compared to the controls (Figure 1C). In Western blot, the desARE3’UTR ERBB2-3 and -30 (Figure 1D) caused significant downregulation of ERBB2 protein expression compared to controls. The desARE3’UTR ERBB2-1, -3, and -30 also caused a significant decrease in ERBB2 transcript expression compared to controls (Figure 1E). The morphology of the destabilized cells shows a ruptured membrane compared to controls (Supplementary Figure S1A). The significant downregulation of ERBB2 at the protein level as determined by immunofluorescence and Western blot was quantified and displayed in Supplementary Figure S1B. We found the increased expression of cleaved caspases 3 and 9 in the desARE3’UTR ERBB2-1, -3, -30-treated cells (Supplementary Figure S2D, E, F, G, H), and treated cells show significant inhibition of migration as demonstrated by significantly impaired wound healing (Supplementary Figure S2I, J). Taken together, these results demonstrate that the engineered destabilized 3’UTR ARE of ERBB2 targeted, destabilized, and degraded the ERBB2 transcript and protein expression and induced increased apoptosis and reduced migration and outperformed trastuzumab in the NCI-H1975 model of the deadly EGFR T790M non-small-cell lung cancer.

Having established that the ERBB2-destabilizing constructs work and degrade the ERBB2 transcript and protein expression, we turned our attention to the devastating clinical problem of ERBB2+ trastuzumab-resistant cancers. Trastuzumab has improved overall survival outcomes for ERBB2+ breast cancer. However, more than 25% of early-stage ERBB2+ breast cancer patients develop resistance to trastuzumab and more than 75% late-stage ERBB2+ breast cancers are resistant to trastuzumab even if given in combination with anthracyclines. More so, there is no effective treatment for drug resistance in ERBB2+ breast cancer tumor relapse. To ascertain if the novel engineered destabilized 3’UTR of ERBB2 will successfully target ERBB2 in trastuzumab-resistant breast cancer cells and control its aggressiveness, we experimented with the BT474 clone 5 (Reuter and Matthews, 2010; Torabi et al 2021) The ERBB2+ trastuzumab-resistant breast cancer cell line was developed by prolonged exposure to trastuzumab. We transfected these cells with the ERBB2 3’UTR destabilizing constructs, and within 9 days (Figure 1F), we found by immunofluorescence analysis that ERBB2 (stained in red) in the treated cells is diminished compared to the wildtype cells and vector controls (Figure 1F; Supplementary Figure S1E). We confirmed this by Western blotting (Figure 1H) and showed that desARE3’UTR ERBB2-3 significantly degraded ERBB2 expression and desARE3’UTR ERBB2-30 led to more than 70% significant downregulation of ERBB2 protein expression in the ERBB2+ trastuzumab-resistant breast cancer cells compared to wildtype and vector control cells (Figure 1H; Supplementary Figure S1F). The treated cells (except for those treated with desARE3’UTR ERBB2-4) also showed loss of viability compared to the controls (Figure 1G). The morphology of the trastuzumab-resistant ERBB2+ cancer cells containing the destabilized elements shows shrinking and reduction in cell size compared to the controls (Figure 1F; Supplementary Figure S1D). Transcript analysis by the quantitative reverse transcriptase polymerase chain reaction (qPCR) showed that all the ERBB2 3’UTR-destabilizing constructs (Figure 1I) significantly reduced the ERBB2 transcript, except for desARE3’UTR ERBB2-4 compared to the controls. ERBB2 3’UTR-destabilizing constructs induced increased caspase 3 in the trastuzumab-resistant ERBB2+ cancer cells (Supplementary Figure S1G). Again, this result demonstrates that the engineered destabilizing ERBB2 3’UTR outperformed trastuzumab in the drug-resistant setting.

**FIGURE 2:**
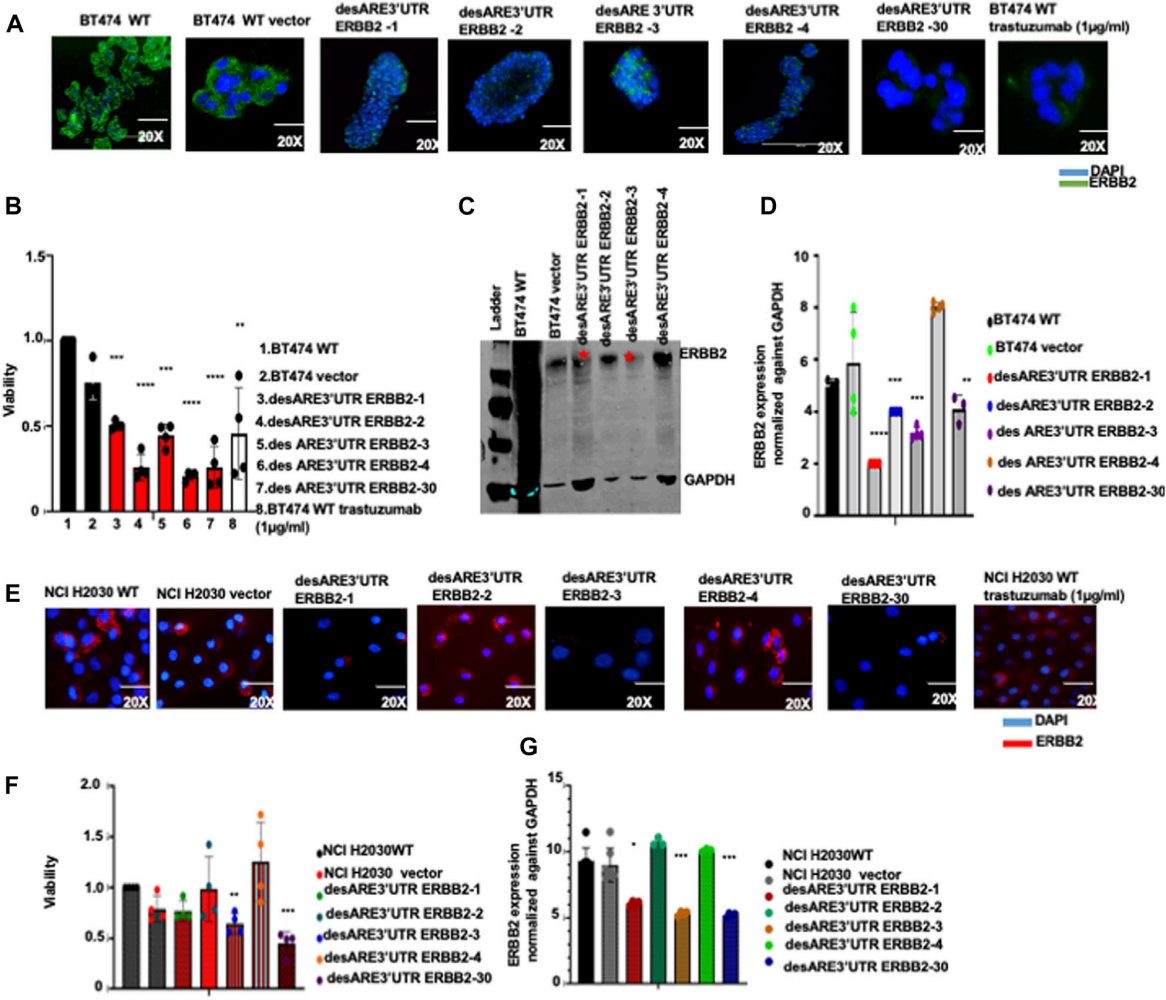
Destabilized 3’UTR of ERBB2 degrades the ERBB2 transcript and leads to loss of expression of oncogenic ERBB2 protein in wildtype ERBB2+ breast cancer cells and in ERBB2-mutated lung cancer cells leading to cell death. **(A)** Immunofluorescence images show ERBB2 expression (stained in green) and nuclei (DAPI-blue) on wildtype BT474, vector, and on BT474 containing constructs desARE3’UTR ERBB2-1, -2, -3, -4, -30, and BT474 wildtype trastuzumab-treated cells (n = 2). Magnification is 20x. **(B)** Bar charts show cell viability of the BT474 wildtype cells, vector, and BT474 cells containing constructs desARE3’UTR ERBB2-1, -2, -3, and -4 (n = 4). Two-tailed T-test (***p-value = 0.0025 WT, vector vs. desARE3’UTR ERBB2-1, ****p-value = 0.00029515 WT, vector vs. desARE3’UTR ERBB2-2, ***p-value = 0.0045 WT, vector vs. desARE3’UTR ERBB2-3, ****p-value = 0.000102 WT, vector, desARE3’UTR ERBB2-4, ***p-value = 0.00120 WT, vector, vs. desARE3’UTR ERBB2-30, **p-value = 0.027 WT, vector vs. BT474 WT trastuzumab treated. **(C)** Western blots show ERBB2 and GAPDH expression across the wildtype BT474, vector, and on BT474 containing constructs desARE3’UTR ERBB2-1, -2, -3, and -4 (n = 2). **(D)** Bar charts show quantification of the ERBB2 expression normalized against GAPDH by qPCR on BT474 wildtype cells, vector and cells containing constructs desARE3’UTR ERBB2-1, -2, -3, and -30 (n = 2). Two-tailed T-test (****p-value = 0.0003 WT, vector vs. desARE3’UTR ERBB2-1, ***p-value = 0.0021 WT, vector vs. desARE3’UTR ERBB2-2,3, **p-value = 0.013 WT, vector vs. desARE3’UTR ERBB2-30). **(E)** Immunofluorescence images show ERBB2 expression (stained in red) and nuclei (DAPI-blue) on wildtype NCI H2030 cells, vector, and on NCI-H2030 containing constructs desARE3’UTR ERBB2-1, -2, -3, -4, and -30, and trastuzumab-treated cells (n = 2). Magnification is 20x. **(F)** Bar charts show cell viability of the NCI H2030 wildtype cells, vector, and cells containing constructs desARE3’UTR ERBB2-1, -2, -3, -4, and -30 (n = 2). Two-tailed T-test (**p-value = 0.0080 WT, vector vs. desARE3’UTR ERBB2-3, ***P = 0.0024 WT, vector vs. desARE3’UTR ERBB2-30). **(G)** Bar charts show quantification of the ERBB2 expression normalized against GAPDH by qPCR on NCI H2030 wildtype cells, vector, and cells containing constructs desARE3’UTR ERBB2-1, -2, -3, -4, and -30 (n = 2). Two-tailed T-test (*p-value = 0.027 WT, vector vs. desARE3’UTR ERBB2-1, ***p-value = 0.0003 WT, vector vs. desARE3’UTR ERBB2-3, ***p-value = 0.0001 WT, vector vs. desARE3’UTR ERBB2-30.

To further validate this, we transfected the four clones (desARE3’UTR ERBB2-1, desARE3’UTR ERBB2-2, desARE3’UTR ERBB2-3, and desARE3’UTR ERBB2-4) of the engineered destabilized 3’UTR ARE of ERBB2 into ERBB2+ BT474 cells. Within 4 days (Figure 2A; Supplementary Figure S1I), we observed by immunofluorescence that ERBB2 protein (stained green) expression has been significantly decreased in the cancer cells containing the destabilizing elements compared to the wildtype and vector control. The morphology of the destabilized cells under a microscope shows a distorted and ruptured membrane compared to the wildtype cells and the vector control (Supplementary Figure S1H). To confirm the significant downregulation of the ERBB2 protein, we performed Western blotting on the wildtype, vector controls cells, and the treated cells (Figure 2C; Supplementary Figure S1J). We found that desARE3’UTR ERBB2-1 and desARE3’UTR ERBB2-3 degraded the ERBB2 protein (Figure 2C; Supplementary Figure S1J) and transcript (Figure 2D) expression. Within 8 days, all the cancer cells transfected with ERBB2 3’UTR-destabilizing elements showed loss of viability and increased caspase 3/7 expression (Supplementary Figure S1K) compared to the wildtype cells and the vector control cells (Figure 2B).

To further demonstrate the generalizability of the novel engineered 3’UTR destabilization of ERBB2 oncogene, we extended this work into the NCIH2030 ERBB2-mutated non-small-cell lung cancer cell line and the HCT116 colorectal cancer cell line. We found that the cells containing ERBB2 3’UTR-destabilizing constructs show significant downregulation of the ERBB2 protein (Figure 2D; Supplementary Figure S1M) and ERBB2 transcript (Figure 2G) and loss of viability (Figure 2F) compared to the wildtype and controls. The morphology of the cells containing the destabilizing constructs desARE3’UTR ERBB2-3 and -30 is different compared to the controls (Supplementary Figure S1L) in NCI-H2030, while desARE3’UTR ERBB2-1, -3, and -30 show morphological changes compared to controls in HCT116 (Supplementary Figure S1N). The desARE3’UTR ERBB2 - 3 and desARE3’UTR ERBB2 - 30 degraded the ERBB2 transcript in HCT116 cells (Supplementary Figure S1O).

Taken together, the findings demonstrate that the novel engineered destabilized 3’UTR of ERBB2 effectively degraded the ERBB2 transcript and protein expression to kill cancer cells in a clinically relevant model of ERBB2+-overexpressing osimertinib-resistant EGFR T790M non-small-cell lung cancer, in ERBB2-mutated non-small-cell lung cancer, in ERBB2+-expressing colorectal cancer cell line, in difficult-to-treat ERBB2+ trastuzumab-resistant breast cancer cell line, and in wildtype ERBB2+-expressing breast cancer.

### Transcriptome analysis shows that engineered destabilized 3’UTR ERBB2 degraded ERBB2 and its interactome through the designed nonsense-mediated decay pathway

**FIGURE 3:**
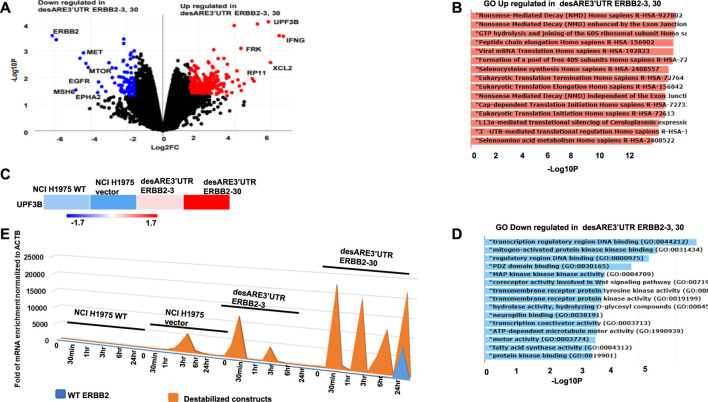
Gene expression analysis of the destabilized 3’UTR of ERBB2 cancer cells show degradation of the ERBB2 transcript and worked as designed through upregulated mRNA decay and engineered 3’UTR translational regulation. **(A)** Volcano plot shows genes downregulated (blue), and genes upregulated (red) in desARE3’UTR ERBB2-3 and -30 compared to wildtype and vector controls. **(B)** Horizontal bar charts show the Gene Ontology (GO) theme upregulated in desARE3’UTR ERBB2-3 and -30 compared to wildtype and vector controls. **(C)** Horizontal bar chart shows UPF3B expression in wildtype NCI-H1975, vector, and desARE3’UTR ERBB2-3 and -30. **(D)** Horizontal bar chart shows Gene Ontology (GO) downregulated in desARE3’UTR ERBB2-3 and -30 compared to wildtype and vector controls. **(E)** Chart shows time-dependent chase of both destabilized construct transcript (orange) and WT ERBB2 mRNA (blue) in the same cells.

To validate at the transcriptome level that the engineered destabilized 3’UTR of ERBB2 degraded ERBB2 and its interactome through the designed mechanism incorporated in the ERBB2 3’UTR-destabilizing constructs, we performed RNA sequencing on the ERBB2-expressing EGFR T790M lung cancer cells NCI-H1975 WT, NCI-H1975 vector, and in NCI-H1975 cancer cells containing desARE3’UTR ERBB2-3 and desARE3’UTR ERBB2-30. We found that desARE3’UTR ERBB2-3 and -30 show strong reduction in ERBB2 and EGFR, respectively (Figure 3A). We engineered the constructs to degrade mRNA once the transcript ARE is destabilized through mRNA decay machinery. By gene ontology analysis, we investigated if the mRNA decay mechanism is triggered once the transcript is destabilized and driven by the mRNA cap deadenylating DCP1A promoter. As designed in the constructs, the gene ontology theme of nonsense-mediated decay is most significantly enriched GO biological and molecular function with a *p*-value = 5.2e-^13^ and FDR = 5.3e-^10^, and the genes UPF3B and UPF3A, which are known regulators of nonsense-mediated decay, were implicated (Figures 3A–C). Again, the ontology theme also identified upregulated 3’UTR-mediated translation regulation (*p*-value = 1.6e-^11^ and FDR = 8.7e-^10^) as part of the mechanism implicated in corroborating our engineering design which specifically targeted the ARE on the 3’UTR of ERBB2 (Figure 3B). The GO-downregulated theme implicated transmembrane receptor tyrosine kinase (*p* = 0.0008, FDR = 0.048) to which ERBB2, which is an EGFR family receptor, belongs (Figure 3D). To determine how the destabilizing constructs degrade the endogenous ERBB2 mRNA, we performed a 4SU mRNA labeling pulse-chase experiment on the NCI-H1975 WT, NCI-H1975 vector, and desARE3’UTR ERBB2-3 and -30 at 0 h, 30 mins, 1h, 3h, 6h, and 24h. We collected RNA, made cDNA, and performed RT-qPCR with primers targeting the endogenous ERBB2 (forward and reverse primers for exon 6) as well as primers targeting the vector and RFP (forward) and cloned destabilizing ERBB2 constructs (reverse). ACTB served as a control housekeeping gene. We found that the destabilized construct transcript is 2.5 × 10^4^ overexpressed more than the endogenous ERBB2. These data indicate that the destabilizing ERBB2 transcript is made at such a high amount that the transcription machinery overwrites/outcompetes the endogenous ERBB2 message with the destabilizing one using this approach (Figure 3E). Based on the thorough 4SU mRNA labeling data shown in Figure 3E, the minimum destabilized transcript level required to outcompete/overwrite the endogenous ERBB2 mRNA is approximately 6 × 10^3^. Using the data from the mRNA pulse-chase time-dependent 4SU experiment, we calculated the half-life of the wildtype ERBB2 and destabilized ERBB2 using one phase decay equation. We found that the half-life of WT ERBB2 approximately 1.5 hrs and the destabilized construct transcript approximately 6 hrs. Indeed, the transcriptome analysis confirmed that these engineered constructs reliably targeted ERBB2 and worked as the engineered constructs were designed to function through mRNA-mediated decay driven by the decapping DCP1A promoter to trigger nonsense-mediated mRNA decay and exonucleases, deadenylases to overwrite/outcompete, and degrade the ERBB2 transcript.

### Engineered destabilized 3’UTR significant downregulation of ERBB2 is sequence specific and triggers exonucleases XRN1 and CNOT1 to degrade the ERBB2 transcript

**FIGURE 4:**
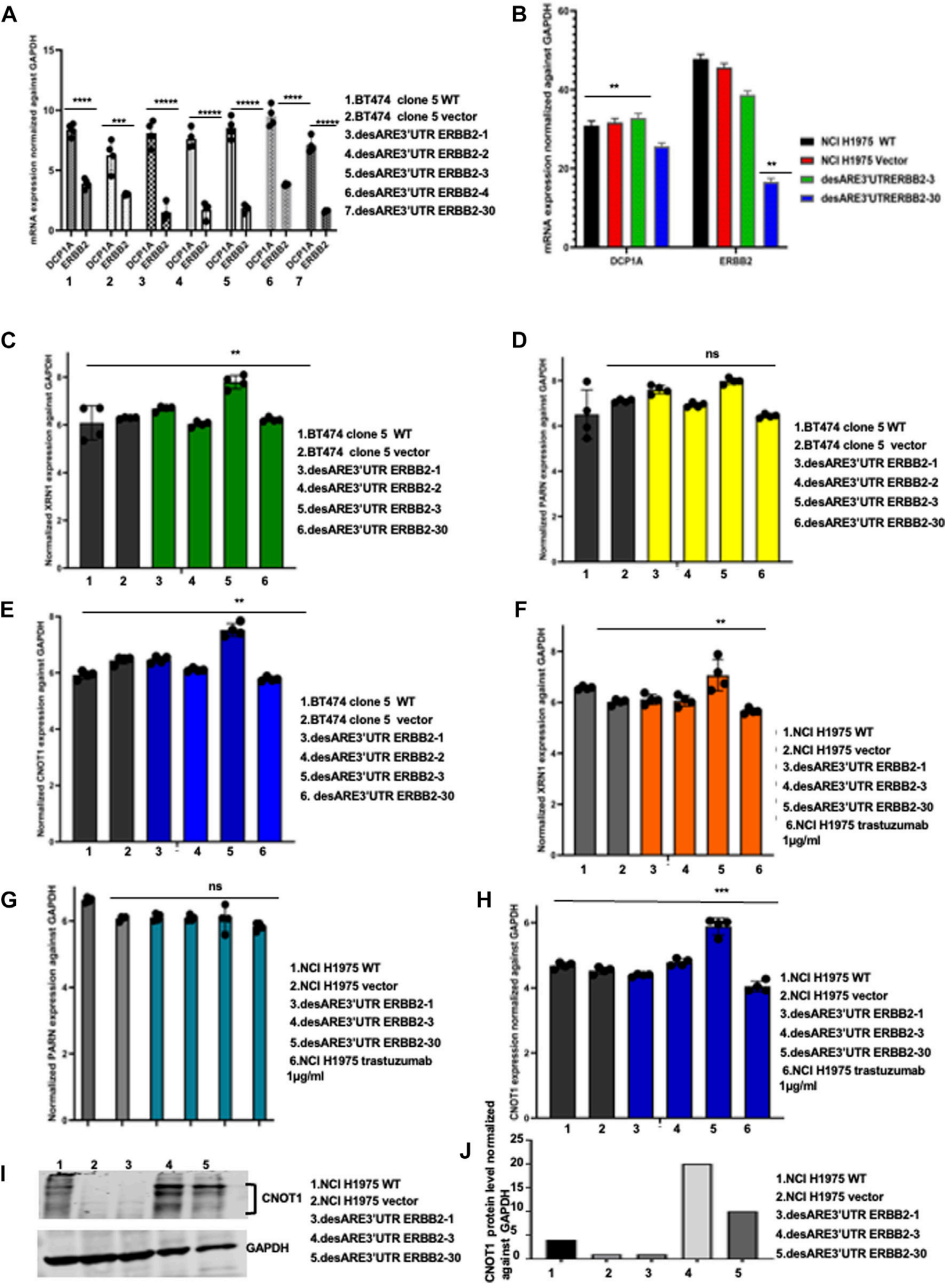
Engineered destabilized 3’UTR of ERBB2 is sequence specific in degrading the ERBB2 transcript through XRN1 and CNOT1. **(A)** Bar charts show mRNA expression of DCP1A and ERBB2 normalized against GAPDH in BT474 clone 5 WT, vector, desARE3’UTR ERBB2-1, -2, -3, and -4 and 30 (n = 2). T-test (****p = 0.0001 ERBB2 vs. DCP1A BT474 clone 5 WT), (***p = 0.0012 ERBB2 vs. DCP1A vector), (*****p = 0.00001 ERBB2 vs. DCP1A desARE3’UTR ERBB2-1), (*****p = 0.00003 ERBB2 vs. DCP1A desARE3’UTR ERBB2-2), (*****p = 0.000025 ERBB2 vs. DCP1A desARE3’UTR ERBB2-3), (****p = 0.00013 ERBB2 vs. DCP1A desARE3’UTR ERBB2-4), (*****p = 0.000016 ERBB2 vs. DCP1A desARE3’UTR ERBB2-30). **(B)** Bar charts show the RNA Seq expression of DCP1A in NCI-H1975 WT, vector, desARE3’UTR ERBB2-3 and -30. T-test (****p = 0.0128 ERBB2 vs. DCP1A NCI-H1975 WT), (**p = 0.011 ERBB2 vs. DCP1A vector), (p = ns ERBB2 vs. DCP1A desARE3’UTR ERBB2-3), (**p = 0.001 ERBB2 vs. DCP1A desARE3’UTR ERBB2-30). **(C)** Bar charts show the mRNA expression of XRN1 normalized against GAPDH in BT474 clone 5 WT, vector vs. desARE3’UTR ERBB2-1, -2, -3, -4, and -30, **p-value = 0.002. Two-tailed t-test (n = 2). **(D)** Bar charts show the mRNA expression of PARN normalized against GAPDH in BT474 clone 5 WT, vector vs. desARE3’UTR ERBB2-1, -2, -3, -4, and -30, *p*-value = ns, two tailed t-test (n = 2). **(E)** Bar charts show the mRNA expression of CNOT1 normalized against GAPDH in BT474 clone 5 WT, vector vs. desARE3’UTR ERBB2-1, -2, -3, -4, and -30, **p-value = 0.001, two tailed t-test (n = 2). **(F)** Bar charts show the mRNA expression of XRN1 normalized against GAPDH in NCI-H1975 WT, vector vs. desARE3’UTR ERBB2-1, -2, -3, -4, and -30 and trastuzumab-treated cells **p-value = 0.004, two tailed t-test (n = 2). **(G)** Bar charts show the mRNA expression of PARN normalized against GAPDH in NCI-H1975 WT, vector vs. desARE3’UTR ERBB2-1, -2, -3, -4, and -30 and trastuzumab-treated cells, *p*-value = ns, two-tailed t-test (n = 2). **(H)** Bar charts show the mRNA expression of CNOT1 normalized against GAPDH in NCI-H1975 WT, vector vs. desARE3’UTR ERBB2-1, -2, -3, -4, and -30 and trastuzumab-treated cells, *p*-value = 0.0002, two tailed t-test (n = 2). **(I)** Western blot showing CNOT1 and GAPDH protein expression in NCI-H1975 WT, vector, desARE3’UTR ERBB2-1, -3, and -30. **(J)** Bar chart show quantification of CNOT1 protein expression normalized against GAPDH in NCI-H1975 WT, vector, desARE3’UTR ERBB2-1, -3, and -30.

To ascertain the molecular details of how the degradation of ERBB2 is achieved (Figures 1B,D,F,H; 2A,C,E), we designed the engineered destabilized 3’UTR of ERBB2 to be driven by the mRNA decapping enzyme DCP1A promoter. To understand if the DCP1A promoter contributed to ERBB2 degradation through mRNA decapping upon the introduction of the engineered destabilizing 3’UTR ARE of ERBB2 into the breast and lung cancer cells, we performed qRT-PCR and showed that DCP1A promoter expression did not change upon destabilization of the ERBB2 transcript compared to the controls (Figure 4A). More so, DCP1A expression from RNA seq (Figure 4B) shows that DCP1A does not increase in the destabilized constructs compared to control. This finding suggests that the engineered destabilized 3’UTR ARE sequence only explains the degradation of ERBB2. To understand the deadenylation and cleaving proteins involved in this process and if there is a preference for 5’–3’ or 3’–5’ deadenylation and cleavage, we performed a qRT-PCR on the 5’–3’ cleaving enzyme XRN1 and 3’–5’ deadenylase PARN and CNOT1 both on the BT474 clone 5 and the NCI-H1975 containing the destabilized constructs compared to the controls. In the BT474 clone 5 containing the destabilized 3’UTR ARE of ERBB2, we found that the XRN1 expression is significantly elevated in the desARE3’UTR ERBB2-3 (Figure 4C), which completely degraded the ERBB2 protein in Figure 1F; Figure 1H. The PARN expression is not elevated (Figure 4D). The CNOT1 expression is significantly elevated in desARE3’UTR ERBB2-3 (Figure 4E). We found similar results in NCI-H1975 transduced with the destabilizing constructs of the 3’UTR of ERBB2. XRN1 is significantly elevated in desARE3’UTR ERBB2-30 (Figure 4F); also, CNOT1 is significantly elevated in the desARE3’UTR ERBB2-30 (Figure 4H). desARE3’UTR ERBB2-30 showed the most significant downregulation of ERBB2 in Figure 1B. Again, the PARN expression is not elevated in NCI-H1975 (Figure 4G) as seen in the BT474 clone 5 (Figure 4D). Finally, to prove the increased CNOT1 protein expression in the destabilized cells, we performed a Western blot and show that CNOT1 is highly expressed in the cells carrying desARE3’UTR ERBB2-3 and -30 (Figures 4I,J). *In vivo*, we found a significant downregulation of ERBB2 in animals bearing tumors treated with desARE3’UTR ERBB2-1, -3, and -30 and increased expression of UPF3B, XRN1, and CNOT1 (Supplementary Figure S14A–H). These results suggest a general mechanism that both 5’–3’ and 3’–5’ deadenylases, cleaving enzymes, and nonsense-mediated decay are involved in the degradation of the ERBB2 once the 3’UTR of ERBB2 is destabilized.

### Destabilized 3’UTR degradation of ERBB2 in the osimertinib- and trastuzumab-resistant cancer cell lines leads to loss of kinases implicated in breast and lung cancer drug resistance

The signaling cascade of ERBB2 is mediated by kinases and as such the resistance to anti-ERBB2 is concomitant with the increased activity of kinase that helps the drug-resistant cancers to proliferate and metastasize. Osimertinib EGFR T790M resistance arises due to upregulation of EGFR-dependent kinases and EGFR-independent pathway. We explored that destabilization of ERBB2 in this non-small-cell lung cancer line does, indeed, control these implicated kinases and proteins. We found that EGFR, MET, and MAPK kinases implicated in the EGFR dependent pathway of osimertinib resistance were all downregulated (Supplementary Figure S2A, B; Supplementary Table S2). More so, the EGFR-independent pathways such as MTOR, CNNTB1, EPHA2, and NOTCH (Supplementary Figure S2C) were downregulated upon destabilization of ERBB2. This finding points to the superiority of this approach in controlling drug resistance driven by ERBB2 and its widespread interactome, such that when we control ERBB2, we controlled all the pathways that are dependent on it.

**FIGURE 5:**
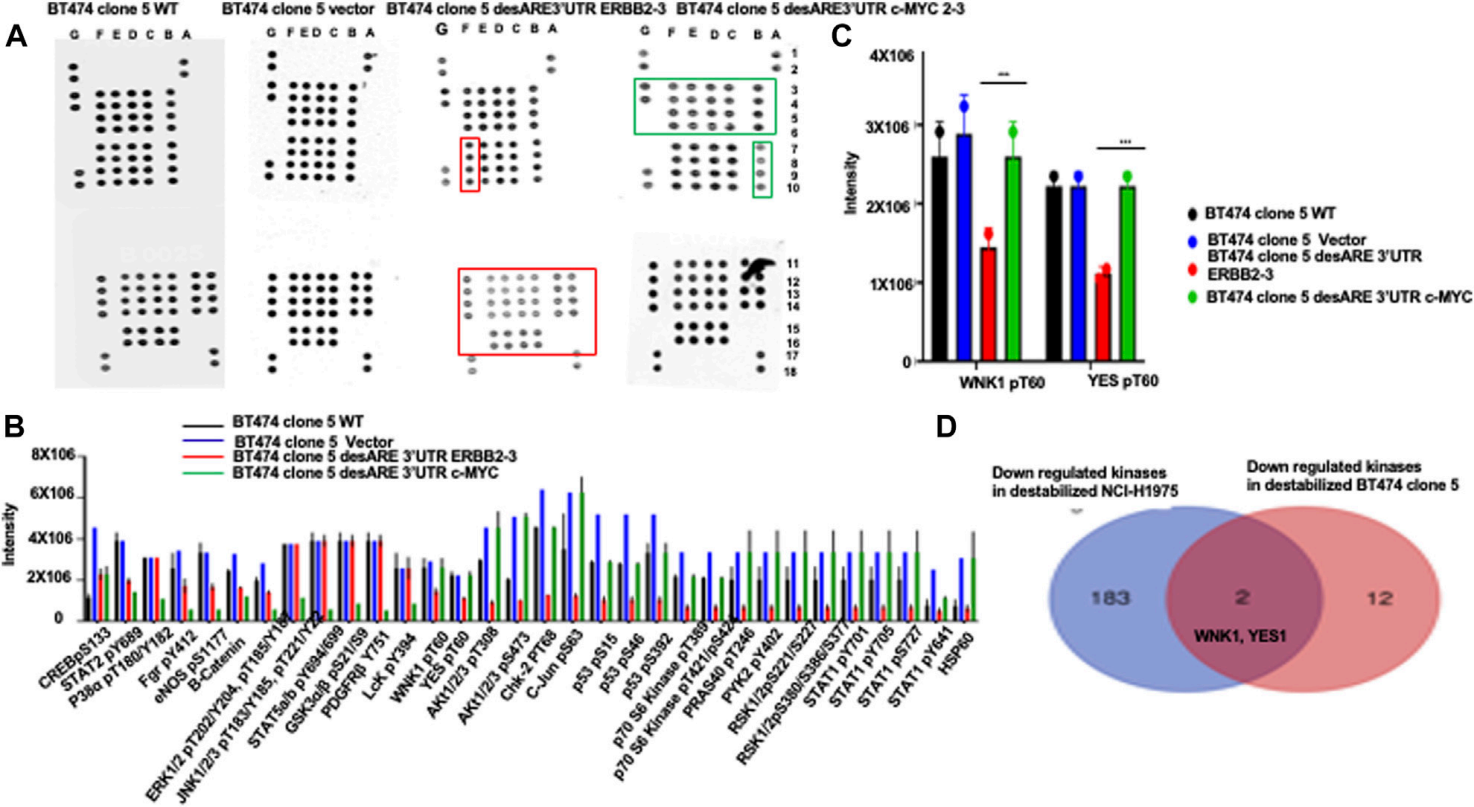
Engineered destabilized 3’UTR of ERBB2 degrades specific kinases implicated in ERBB2+ trastuzumab-resistant breast cancer and in ERBB2-expressing osimertinib-resistant EGFR790M non-small-cell lung cancer cells. **(A)** Dot blots show the phospho-kinase array pattern of the BT474 clone 5 wildtype, BT474 clone 5 vector, BT474 clone 5 desARE3’UTR ERBB2-3, and BT474 clone 5 desARE3’UTR c-MYC 2–3. The dot marked in the red box shows unique kinases downregulated only in BT474 clone 5 desARE3’UTR ERBB2-3 and the dot marked in green boxes shows unique kinases downregulated only in BT474 clone 5 desARE3’UTR c-MYC 2-3. Each kinase is spotted twice. **(B)** Bar chart shows the intensity of the phospho-kinase array dot blot for the BT474 clone 5 wildtype (black bar), BT474 clone 5 vector (blue bar), BT474 clone 5 desARE3’UTR ERBB2-3 (red bar), and BT474 clone 5 desARE3’UTR c-MYC 2-3 (green bar). **(C)** Bar charts show YES1 pT60 and WNK1 pT60 kinases specifically downregulated in BT474 clone 5 desARE3’UTR ERBB2-3 in red bar. Controls BT474 clone 5 wildtype (black bar), BT474 clone 5 vector (blue bar), and BT474 clone 5 desARE3’UTR c-MYC 2-3 (green bar). T-test (***p-value = 0.00413, WT, vector vs. desARE3’UTR ERBB2-3 for YES and WNK1). **(D)** Venn diagram shows kinases downregulated in destabilized desARE3’UTR ERBB2-3 and -30 NCI- H1975 (p = 0.02) blue (from RNA Seq), and in destabilized desARE3’UTR ERBB2-3 BT474 clone 5 (red-kinase array) and the shared kinases found downregulated in destabilized ERBB2 in both resistant cell lines WNK1 and YES1 in pink.

To ascertain if the destabilized 3’UTR of ERBB2 degradation of ERBB2 led to loss of kinases known to contribute to ERBB2+ trastuzumab resistance, we performed a phosphorylation kinase array assay with protein extracts from BT474 clone 5 WT, BT474 clone 5 vector, BT474 clone 5 desARE3’UTR ERBB2-3, and desARE3’UTR c-MYC 2–3 (a specificity control for ERBB2 destabilization) (Figures 1F–H; Figures 5A,B). We found that kinases such as AKT1/2/3 pT308, AKT1/2/3 pS473, Chk-2 pT68, c-JUN pS63, P53 pS46,P53pS15, P53 pS392, P70 S6 kinase pT389, P70 S6 kinase pT421/pS424, PRAS 40 pT246, PYK2 pY402, RSK1/2 pS221/pS227, RSK1/2/3 pS380/pS386,pS377, STAT1pY701, STAT2 pY705, STAT2 pS727, STAT3 pS727, STAT6 pY641, and HSP60 marked with a red box in the lower panel of desARE3’UTR ERBB2-3 were all downregulated upon the degradation of ERBB2 by 3’UTR destabilization compared to controls (Figures 5A,B).

These kinases downregulated have been implicated in mediation of trastuzumab resistance in breast cancer, both from clinical trials and in cancer cell lines (Kute et al 2004; Zazo et al 2016; Wang et al 2020; Jaykumar et al., 2021). Second, the result revealed ERBB2 is a master regulator controlling a vast array of kinases and transcription factors and that our novel developed technology can control not only the ERBB2 but as well all the signaling cascade and interactome associated with it. We also explored kinases that were lost in desARE3’UTR ERBB2-3 samples but whose expression is relatively similar both in wildtype, vector control, and desARE3’UTR c-MYC 2-3; a specificity control for ERBB2 destabilization, we found the kinases YES pT60 and WNK1 pT60 (Figures 5A–C) marked with a red box in the upper panel of desARE3’UTR ERBB2-3. To find the universal common kinases that mediate drug resistance both in osimertinib-resistant EGFR T790M lung cancer and trastuzumab-resistant breast cancer that are downregulated upon our destabilization of ERBB2, we triaged all the kinases downregulated in ERBB2 destabilized NCI-H1975 (Supplementary Table S2) found by RNA Seq with kinases downregulated in ERBB2 destabilized BT474 clone 5 (Figures 5A–C) found by the kinase array. Indeed, we found that WNK1 and YES1 is the common shared kinase by these two cancer cell lines that mediates drug resistance in them amongst other (Figure 5D).

To assess the specificity of the kinases downregulated through the destabilization of the 3’UTR of ERBB2 is unique to ERBB2 and not to other oncogenes such as c-MYC, we performed a phosphorylation kinase array assay on the protein extract of the BT474 clone 5 destabilized with desARE3’UTR MYC 2-3. We found in Figures 5A,B, the last panel marked with green box, that the kinases lost by the destabilization of c-MYC is unique to c-MYC and that kinases lost by destabilization of ERBB2 is unique to ERBB2, proving surprisingly that the destabilization of the ARE 3’UTR of oncogenes is unique and specific and a targeted molecular therapy.

**FIGURE 6:**
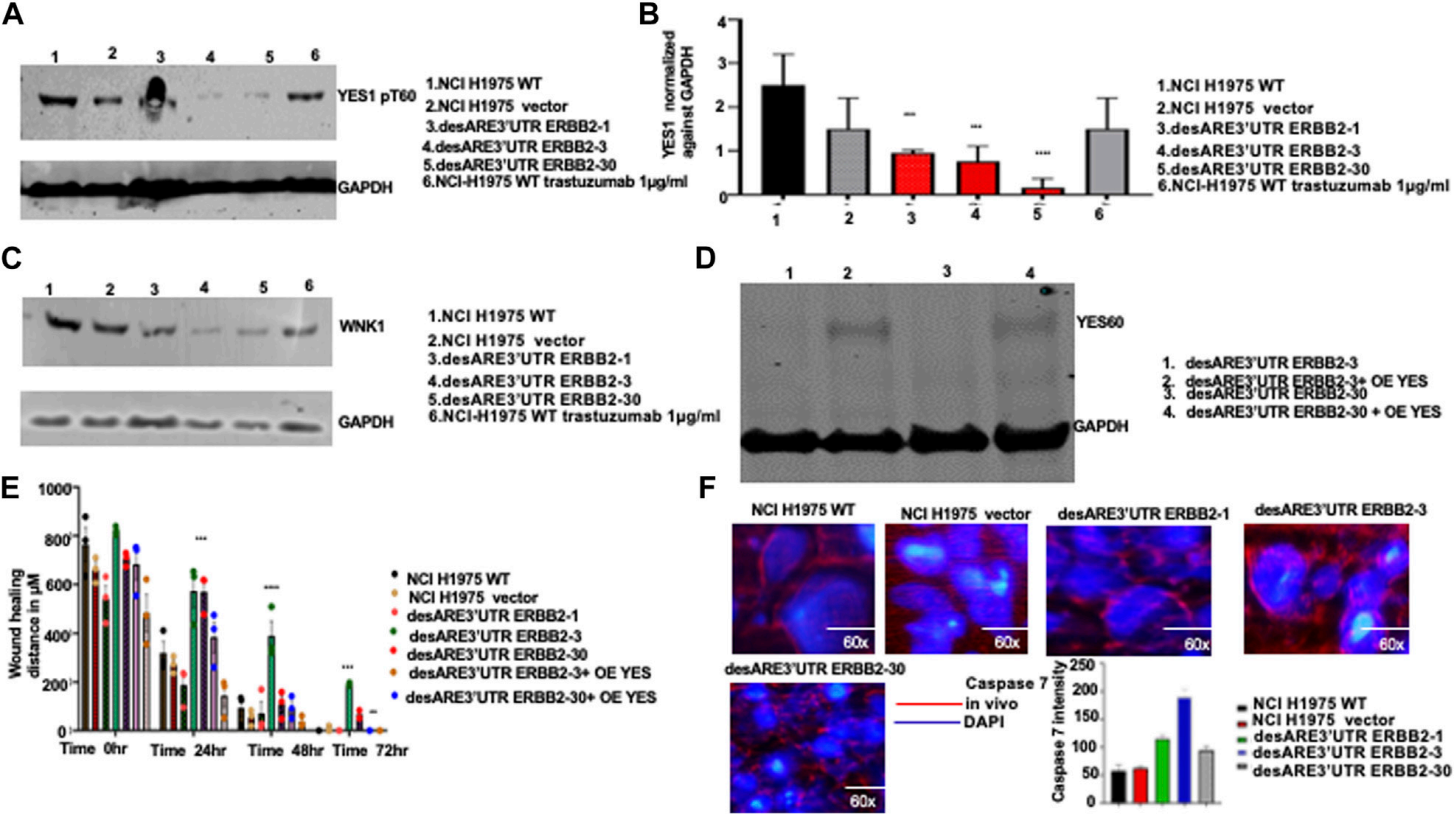
Validation of the engineered destabilized 3’UTR of ERBB2 degradation of YES1 and WNK1 and inhibition of cancer cell migration and induction of caspase 7 *in vivo* in treated tumor-bearing mice. **(A)** Western blot show YES1 and GAPDH expression across the NCI-H1975 wildtype cells, vector, cells containing constructs desARE3’UTR ERBB2-1, -3, and -30, and wildtype trastuzumab-treated NCI-H1975 cells (n = 2). **(B)** Bar chart shows quantification of the YES1 expression normalized against GAPDH on NCI-H1975 wildtype cells, vector, and cells containing constructs desARE3’UTR ERBB2-1, -3, and -30 (n = 2). T-test (***p-value = 0.0021, WT, vector vs. desARE3’UTR ERBB2-1, -3, and -30). **(C)** Western blot shows WNK1 and GAPDH expression across the NCI-H1975 wildtype cells, vector, cells containing constructs desARE3’UTR ERBB2-1, -3, and -30 and wildtype trastuzumab-treated NCI-H1975 cells. **(D)** Western blot shows YES1 and GAPDH expression across the NCI-H1975 destabilized with constructs desARE3’UTR ERBB2-3 and -30 (lanes 1 and 3) and over expressed with YES1 in lanes 2 and 4. **(E)** Bar charts show the wound healing closing of NCI-H1975 wildtype, vector, desARE3’UTR ERBB2-1, -3, -30, and desARE3’UTR ERBB2-30,30 overexpressed with YES1 from 0 h to 72 hrs. At 24hrs ***p = 0.00025 WT, vector vs. desARE3’UTR ERBB2-3,30. At 48 hrs ****p = 0.000018 WT, vector vs. desARE3’UTR ERBB2-3 and at 72 hrs ***p = 0.0003 WT, vector vs. desARE3’UTR ERBB2-3 and **p = 0.002 WT, vector vs. desARE3’UTR ERBB2-30. **(F)** Pictures depict caspase 7 expression *in vivo* in tumors untreated NCI-H1975 wildtype, vector, desARE3’UTR ERBB2-1, -3, and -30 and quantified in bar charts. Magnification is 60x.

To validate the loss of YES1 pT60 and WNK1 upon the destabilization of the 3’UTR of ERBB2 (Figures 5A–D), we performed a Western blot on desARE3’UTR ERBB2 samples compared to the controls. We found that YES1 pT60 protein expression was decreased in the desARE3’UTR ERBB2-3 and -30 compared to the controls across multiple ERBB2-driven cancer types such as NC-H1975 (Figures 6A,B). The engineered destabilized desARE3’UTR ERBB2-1, -3, -30 degraded WNK1 (Figure 6C). Taken together, we demonstrate that by destabilizing ERBB2, we controlled and downregulated YES1 and WNK1 that are well-known causes of drug resistance in ERBB2+ trastuzumab drug-resistant breast cancer and in osimertinib-resistant EGFR T790M lung cancer, respectively (Kute et al 2004; Zazo et al 2016; Wang et al 2020; Jaykumar et al., 2021).

In a gain of function experiment, we overexpressed YES1 using a construct developed by Rosenbluh et al. (2012) in NCI-H1975 desARE3’UTR ERBB2-3 and -30 cells that we degraded and reduced ERBB2 and YES1. We confirmed the overexpression of YES1 by the Western blot (Figure 6D). Next, we performed a wound healing assay comparing the wildtype control, vector, desARE3’UTR ERBB2-1, -3, and -30 and desARE3’UTR ERBB2-3 and -30 with overexpressed YES. We found that (Figure 6E; Supplementary Figure S11) desARE3’UTR ERBB2-3 and -30 OE YES closed their wound at similar time points as the controls. The cells bearing the destabilized desARE3’UTR ERBB2-3 and -30 were unable to close their wounds due to lack of ERBB2 and YES (Figure 6E; Supplementary Figure S11) and this triggered caspase 7 (Figure 6F). This finding confirms ERBB2 and its downstream YES kinase control resistance in NCI-H1975 and that this novel approach we developed is superior in controlling ERBB2 pervasive oncogene signal and their downstream YES kinases that are involved in drug resistance which osimertinib and trastuzumab cannot control.

### The engineered destabilized 3’UTR of ERBB2 outperforms ShRNAi and genome-scale CRISPR in controlling the ERBB2 transcript in ERBB2-expressing colorectal cancer HCT116

To evaluate the performance of the engineered destabilized ARE 3’UTR of ERBB2 in controlling the ERBB2 transcript and downstream kinases in comparison to ShRNAi and genome-scale CRISPR, we analyzed the publicly available database for ShRNAi and CRISPR (Vizeacoumar et al., 2013; Hart et al., 2015; Martin et al., 2017) in HCT116, a colorectal carcinoma cell, an ERBB2-expressing cancer. We found (Table 1) that ShRNAi and CRISPR across multiple studies were unable to downregulate the ERBB2 transcript in HCT116 and its associated kinases, whereas desARE3’UTR ERBB2 degraded the ERBB2 transcript in this study (Supplementary Figure S1N, O) as well as YES1 (Figures 5, 6). Hart et al. (2015) suggested that lack of TP53^S241F^ EGFR dependency in HCT116 prevented erlotinib and ERBB2 and EGFR sgRNA from controlling ERBB2 in the HCT116 KRAS ^G13D^ cell line. The engineered destabilized ARE 3’UTR of ERBB2 is agnostic to these mutations and reliably controlled and degraded the ERBB2 transcript in this cancer. This finding strongly suggests that destabilization of stable oncogene 3’UTR ARE is superior to RNAi and CRISPR in certain genetic mutation settings.

**TABLE 1:** Comparison of ShRNAi, CRISPR, and mRNA 3’UTR destabilization in targeting HER2 in colorectal carcinoma HCT116.

Transcript perturbation method	Publication	Target	Cell line	Outcome	Associated kinases outcome
Genome-wide ShRNA	Vizeacoumar et al. (2013)	EGFR, ERBB2	HCT116	ERBB2 not downregulated	YES1 not downregulated
Genome-wide CRISPR KO	Hart et al. (2015)	EGFR, ERBB2	HCT116	EGFR and ERBB2 not downregulated	
Genome-wide CRISPR KO plus shRNA	Martin et al. (2017)	EGFR, ERBB2	HCT116	EGFR and ERBB2 not downregulated	YES1 not downregulated
desARE3'UTR ERBB2-1 and -3	Awah CU et al., 2022, this Publication	EGFR, ERBB2	HCT116	ERBB2 downregulated	YES1 downregulated

### Engineered destabilized 3’UTR of ERBB2 reprogrammed lung cancer ERBB2 gene expression toward the normal lung epithelial gene expression pattern

**FIGURE 7:**
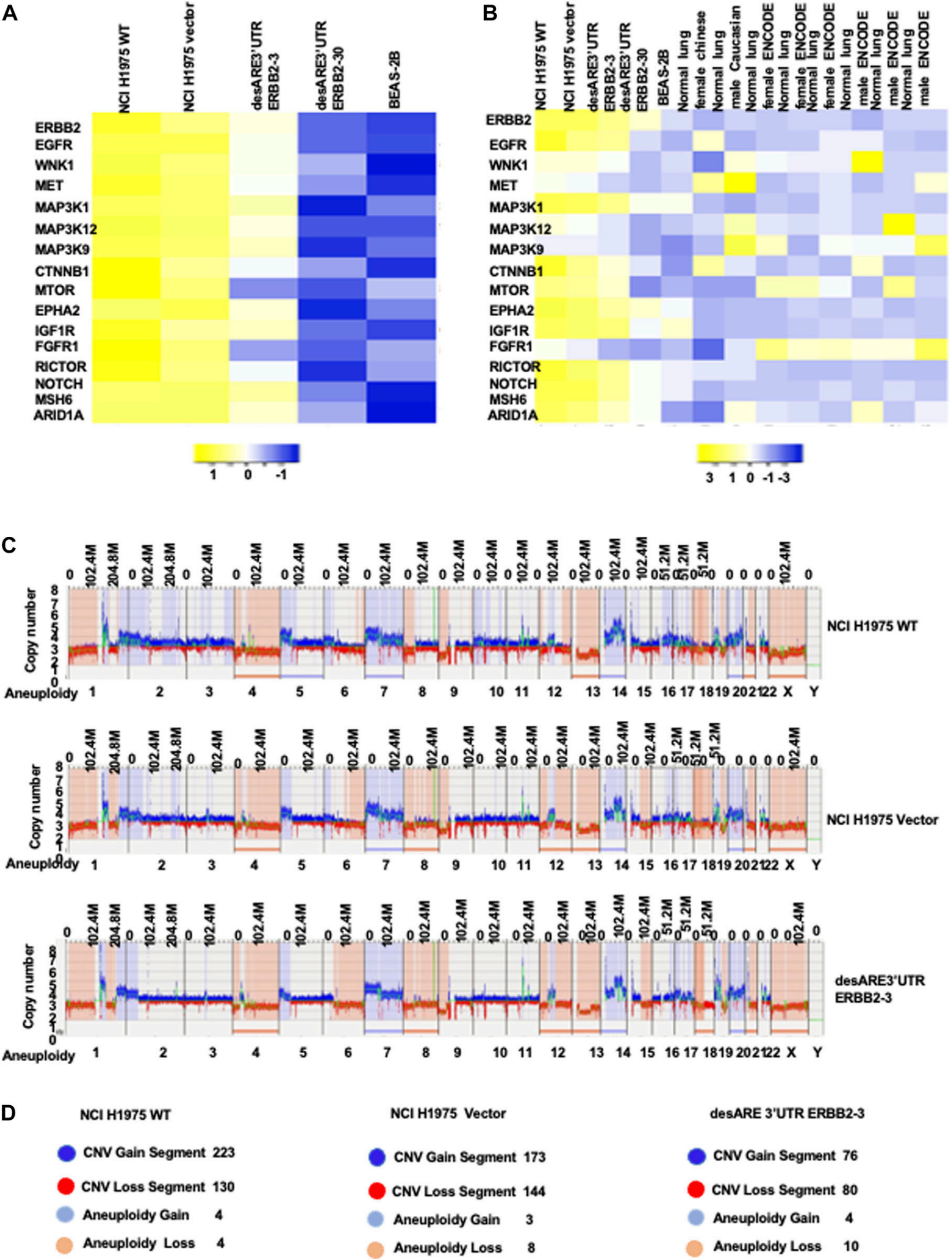
Engineered destabilized 3’UTR of ERBB2 degradation of ERBB2 transcript reprogramed lung cancer ERBB2 gene expression toward normal lung gene expression profile. **(A)** Heat map shows the ERBB2 gene expression profile in wildtype NCI-H1975, vector, desARE3’UTR ERBB2-3 and -30 compared to the BEAS-2B normal human lung epithelial cells. **(B)** Heat map shows the ERBB2 gene expression profile in wildtype NCI-H1975, vector, desARE3’UTR ERBB2 -3 and -30 compared to the BEAS-2B normal human lung epithelial cell line and normal lungs from a Chinese woman non-smoker, normal lung from a Caucasian male, three normal female lungs from ENCODE, and three normal male lungs from ENCODE. **(C)** Genome browser view of whole-genome *de novo* variant assembly between NCI-H1975 wildtype, vector, and desARE3’UTR ERBB2-3. **(D)** Schematic representation of the genomic alteration and their quantification in wildtype NCI-H1975, vector, desARE3’UTR ERBB2-3. Dark blue color- CNV segment gain), red- CNV segment loss), light blue- aneuploidy gain), and light orange- aneuploidy loss (n = 2).

To understand if destabilization of the ERBB2 transcript by 3’UTR engineering restored the lung genetic program toward normal lung epithelial cells, we compared the ERBB2 gene expression profile of wildtype NCI-H1975, vector, desARE3’UTR ERBB2-3 and -30, and BEAS-2B, a normal human lung epithelial cell line (E-MTAB-4729, Supplementary Table S3). We found that the degrading of the ERBB2 transcript by destabilized 3’UTR changed the cancer ERBB2 gene expression almost to near normal lung epithelial cell gene expression program (Figure 7A; Supplementary Table S3). We extended these data to compare if destabilization of the ERBB2 transcript and protein in cancers does reprogram the gene expression of cancers toward the normal human lung gene expression pattern. For this purpose, we obtained the RNA Seq of diverse normal human lung individuals (normal Chinese female non-smokers, SRR1797221, normal Caucasian male, normal female (3), and normal male (3) ENCODE data Supplementary Table S3). Indeed, in Figure 7B, we found that desARE3’UTR ERBB2-30 reprogrammed the cancer ERBB2 gene expression to the normal pattern as in healthy humans. These data strongly suggest that 3’UTR destabilization of the oncogene transcript is safe and achieves the restoration of normal gene expression patterns.

### Genome-wide optical genome mapping shows that engineered destabilized 3’UTR ERBB2 *de novo* and rare variant assembly are similar with the parental cell line

To rule out that our engineered destabilization of the 3’UTR of ERBB2 did not cause severe genome alterations and genome rearrangements in the engineered cells compared to the wildtype and control cells, we used the ultrasensitive technique of optical genome mapping (Lührmann et al., 2021) and found that the cells carrying the constructs desARE3’ERBB2-3 showed almost the same *de novo* and rare variant assembly compared to the wildtype cells and the vector (Figure 7C; Supplementary Figure S9A, 10A). We found that desARE3’UTR ERBB2-3 has lesser copy number variation segment gain, lesser copy number variation segment loss, and more aneuploidy loss than the wildtype and vector. This result can be interpreted as the destabilizing construct desARE3’UTR ERBB2 is safe for the cells and had lesser deleterious changes than the wildtype and vector control (Figure 7D). Aneuploidy is a major cause of chromosomal instability and cancer. It is surprising to find that in these results, the engineered constructs destabilized ERBB2 and led to a significant loss of aneuploidy in the cancers carrying the constructs compared to the controls. These results suggest that destabilized 3’UTR degradation of the ERBB2 transcript has anti-cancer activity amongst which is reduction in cancer cell aneuploidy.

### Engineered destabilized 3’UTR of ERBB2 significantly decreased tumor volume *in vivo* with no toxicity or adverse effects

**FIGURE 8:**
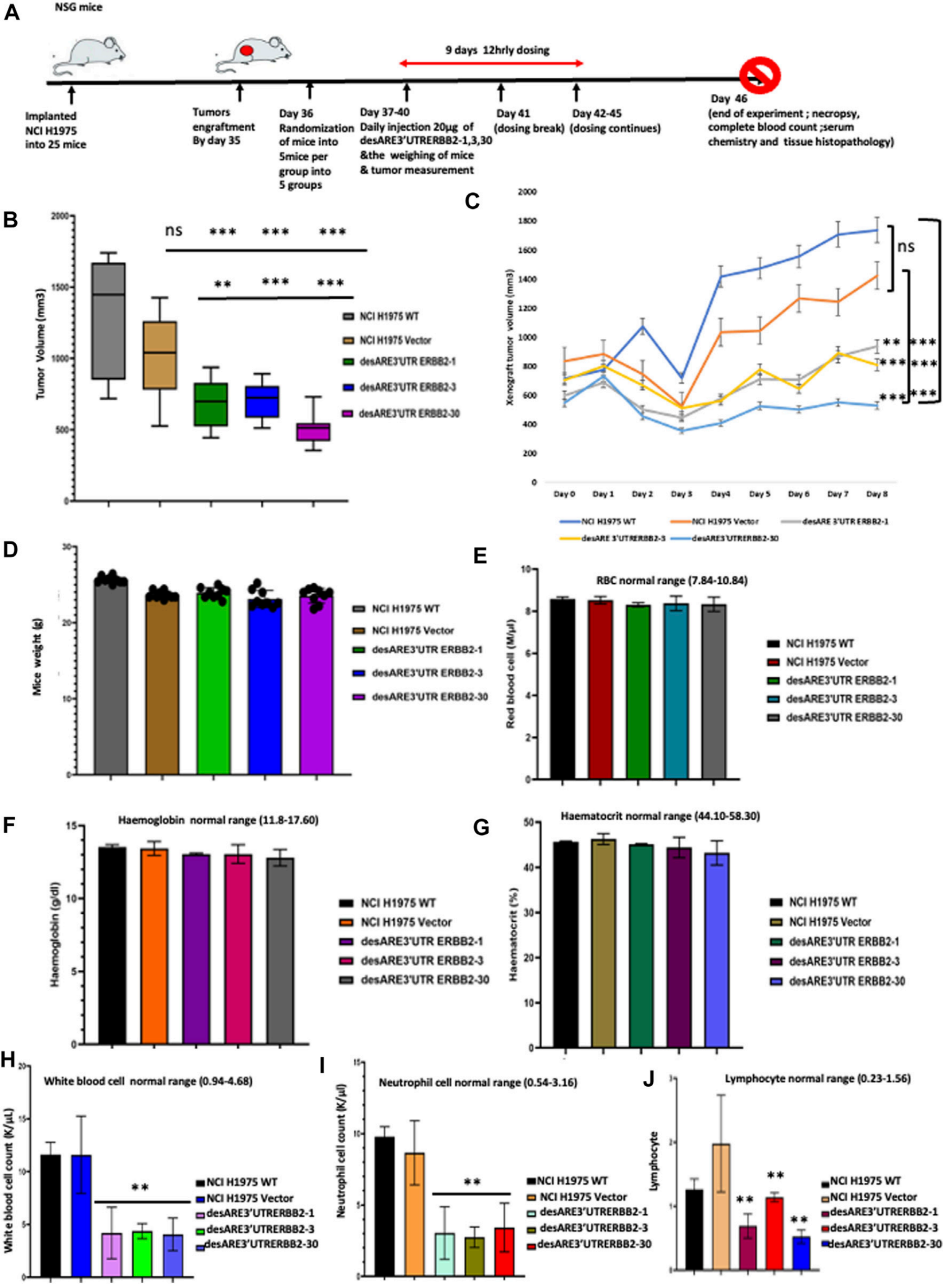
Engineered destabilized 3’UTR of ERBB2 significantly decreased tumor volume *in vivo* with no toxicity or adverse effects. **(A)** Schema depicts outline of animal experiment. A total of 25 NSG female mice were implanted with 5 million NCI-H1975 as a flank xenograft, and after 35 days post implantation, huge tumor engrafts were present. Day 36 post implantation, we randomized the tumors for equal size distribution. Treatment groups were administered 20 µg IP 12 hrly of engineered constructs for 9 days with daily weight and tumor size measurement. Day 46 post implantation, control group tumor sizes have grown so enormous that mice were euthanized, and full blood count, electrolyte, necropsy, and histopathological analysis were performed by an expert pathologist. **(B)** Box plot shows tumor volumes in NCI0H1975 WT (gray), vector (light brown), desARE3’UTR ERBB2-1 (green), desARE3’UTR ERBB2-3 (blue), and desARE3’UTR ERBB2-30 (purple). Two-tailed T-test, *p*-value ns = WT vs. vector, WT vs. desARE3’UTR ERBB2-1, -3, and 30 (***0.0026, ***0.0077, and ***0.004), Vector vs. desARE3’UTR ERBB2-1, -3, and -30 (**0.0251, ***0.0088, and ***0.0031). **(C)** Line plot shows daily tumor growth by comparing NCI-H1975 WT (dark blue), vector (brown) and desARE3’UTR ERBB2-1 (gray), desARE3’UTR ERBB2-3 (yellow), and desARE3’UTR ERBB2- 30. Two-tailed test, *p*-value ns = WT vs. vector, WT vs. desARE3’UTR ERBB2-1, -3, and -30 (***0.0026, ***0.0077, and ***0.004), vector vs. desARE3’UTR ERBB2-1, -3, and -30 (**0.0251, ***0.0088, and ***0.0031). **(D)** Bar charts show weight of mice bearing tumor NCI-H1975 (WT-gray), vector (brown), and mice bearing tumors treated with desARE3’UTR ERBB2-1 (green), desARE3’UTR ERBB2-3 (blue), and desARE3’UTR ERBB2-30 (purple). **(E)** Bar charts shows the red blood cell count of mice bearing tumor NCI-H1975 WT (black), vector (red), and mice bearing tumors treated with desARE3’UTR ERBB2-1 (green), desARE3’UTR ERBB2-3 (blue), and desARE3’UTR ERBB2-30 (gray). **(F)** Bar charts show the hemoglobin count of mice bearing tumor NCI-H1975 WT (black), orange (vector), and mice bearing tumors treated with desARE3’UTR ERBB2-1 (pink), desARE3’UTR ERBB2-3 (magenta), and desARE3’UTR ERBB2-30 (gray). **(G)** Bar charts show the hematocrit level of mice bearing tumor NCI-H1975 WT (black), vector (beige), and mice bearing tumors treated with desARE3’UTR ERBB2-1 (green), desARE3’UTR ERBB2-3 (purple), and desARE3’UTR ERBB2-30 (light blue). **(H)** Bar charts show the white blood cell count of mice bearing tumor NCI-H1975 WT (black), vector (blue) and mice bearing tumors treated with desARE3’UTR ERBB2-1 (pink), desARE3’UTR ERBB2-3 (green), and desARE3’UTR ERBB2-30 (blue) **p = 0.0065 WT, vector vs. desARE3’UTR ERBB2-1, -3, and 30. **(I)** Bar charts show the neutrophil cell count of mice bearing tumor NCI-H1975 WT (black), vector (yellow), and mice treated with desARE3’UTR ERBB2-1 (light green), desARE3’UTR ERBB2-3 (green), and desARE3’UTR ERBB2-30 (red). ***p = 0.0032 WT, vector vs. desARE3’UTR ERBB2-1, 6/3 and 30. **(J)** Bar charts show the lymphocyte cell count of mice bearing tumor NCI-H1975 WT (black), vector (light orange), and mice treated with desARE3’UTR ERBB2-1 (magenta), desARE3’UTR ERBB2-3 (red), and desARE3’UTR ERBB2-30 (blue). **p = 0.0002 desARE3’UTR ERBB2-1.30. **p = 0.0021 desARE3’UTR ERBB2-3.

To validate our findings that the engineered destabilized 3’UTR of ERBB2 degrades ERBB2 *in vivo* and reduces ERBB2-driven tumor growth and volume in a mouse tumor-bearing model, we implanted 25 female NSG mice with 5 million NCI-H1975 cells on the flank and after 35 days, huge tumors engrafted (Figure 8A). On day 36 (Figure 8A), we randomized the mice into five different groups, namely, wildtype, vector, desARE3’UTR ERBB2-1, desARE3’UTR ERBB2-3, and desARE3’UTR ERBB2-30 with the groups having equal representation of tumor size. We, then, administered the vector and the constructs desARE3’UTR ERBB2-1, -3, and -30 at 20 µg per dose given intraperitoneal 12 hrly for 9 days (Figure 8A). Upon each administration, the mice were weighed, tumor size was measured with calipers, and both length and width were recorded. In addition, the body condition score of the mice was recorded too. We plotted the tumor volumes comparing the wildtype and the mice that received the vector, desARE3’UTR ERBB2-1, -3, and -30. We found that (Figures 8B,C) the tumor volumes were significantly reduced in the mice bearing tumors that received the engineered constructs desARE3’UTR ERBB2-1, -3 and -30 compared to the wildtype and vector and that there were no significant differences between tumor volumes in the wildtype and the vector (Figures 8B,C). This finding strongly validates *in vivo* the already established findings here in which the engineered destabilized 3’UTR of ERBB2 degraded ERBB2 and its interactome and impaired cancer cell growth, size, and migration.

Next, we analyzed the weight of the mice as an early pointer of toxicity. We found that there is no difference between the wildtype, vector, and the mice receiving the engineered desARE3’UTR ERBB2-1, -3, and -30 (Figure 8D). This finding shows that while the tumor volumes in the controls were increasing, the mice that received the engineered constructs had their tumor volume reduced and they gained weight by feeding well which points to the absence of toxicity. To prove at the molecular level that our novel technology had no adverse effects or toxicity on the mice that received them, we performed a complete blood count and complete blood chemistry analysis on the controls (wildtype and vector tumors) and desARE3’UTR ERBB2-1, -3, and -30 mice (Supplementary Table S4). We found that there is no change in the red blood cell count (Figure 8E), hemoglobin (Figure 8F), and hematocrit (Figure 8G), and this points to the fact that the engineered constructs are safe and do not cause red blood cell dyscrasias. We analyzed the white blood cells, neutrophils, and lymphocytes. We found that white blood cells, neutrophils, and lymphocytes (Figures 8H–J) were significantly elevated in the wildtype and vector mice, whereas desARE3’UTR ERBB2-1, -3, and -30 restored the white blood cells, neutrophils, and lymphocytes to the normal levels. This finding confirms that the engineered constructs while reducing the tumor volume by degrading ERBB2 restores the white blood cells, neutrophils, and lymphocytes to the normal level. The high level of the white blood cells, neutrophils, and lymphocytes in the controls shows that the immune cells in those mice are elevated due to the tumor size increase.

Next, we studied the complete blood chemistry of the tumor-bearing control mice and those receiving desARE3’UTR ERBB2-1, -3, and -30. In the kidney function analysis, we found no increase in blood urea nitrogen and creatinine levels (Supplementary Figure S12A, B). The liver function test shows no increase in albumin (Supplementary Figure S12E) and globulin (Supplementary Figure S12F) in controls and tumor-bearing constructs. The alkaline phosphatase levels are low across the control and tumor-bearing mice treated with the engineered destabilized constructs (Supplementary Figure S12C). The aspartate amino transferase level (Supplementary Figure S12D) was elevated across all mice bearing tumors except for the mice receiving desARE3’UTR ERBB2-1. The blood glucose (Supplementary Figure S12H), cholesterol (Supplementary Figure S12I), triglycerides (Supplementary Figure S12J), and total bilirubin (Supplementary Figure S13D) levels are normal between the controls and the tumor-bearing mice receiving the engineered constructs. These results strongly show that there is no abnormal liver, bone, pancreas, and gall bladder function in the mice receiving the engineered destabilized constructs.

**FIGURE 9:**
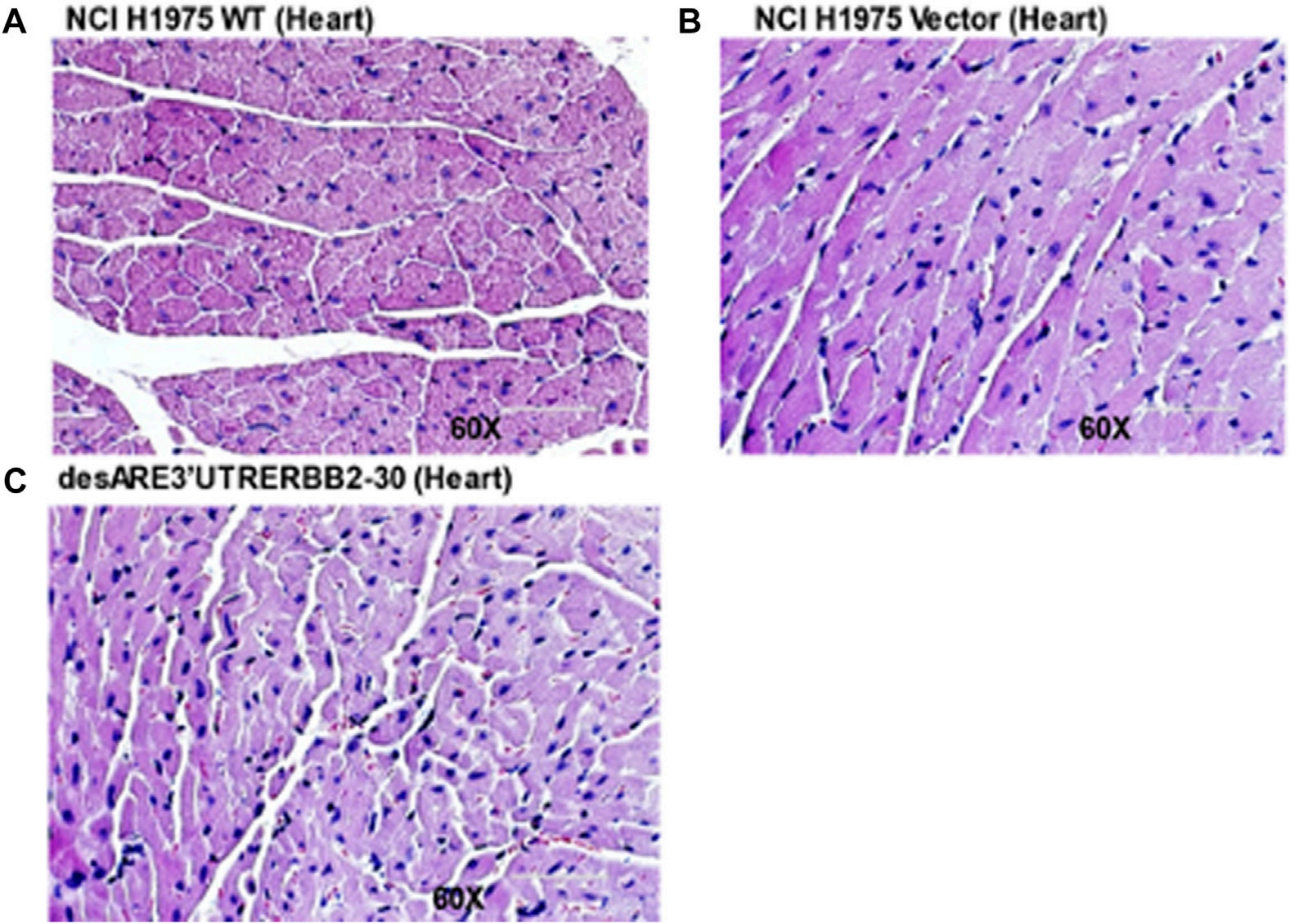
H & E stain of cardiac tissues of the untreated and treated tumor bearing mice. **(A)** H & E stain of the cardiac tissue of the WT (magnification 60x). **(B)** H& E stain of the cardiac tissue treated with the vector (magnification 60x). **(C)** H& E stain of the cardiac tissue treated with the desARE3’UTR ERBB2-30 (magnification 60x).

Furthermore, we examined the blood electrolyte levels by comparing the controls and the tumor-bearing mice receiving the constructs. We found that the calcium (Supplementary Figure S12G), blood CO_2_ (Supplementary Figure S12K), sodium (Supplementary Figure S13A), potassium (Supplementary Figure S13B), and chloride (Supplementary Figure S13C) levels were all normal. Trastuzumab is known to cause cardiac toxicity. We evaluated by H &E staining of the cardiac tissues if our constructs affected cardiac tissue morphology between the treated and untreated animals bearing tumors. We found no observable histological changes in the cardiac tissues (Figures 9A–C).

Taken together, we have validated *in vivo* that the engineered destabilized 3’UTR of ERBB2 significantly reduced the tumor volume in a deadly drug-resistant tumor model with no abnormal blood, liver, kidney, bone, pancreas, gall bladder, and electrolyte imbalance. These findings strongly suggest that the therapy is safe and can be rapidly translated to human cancers. Finally, we stained the xenograft slides of WT mice tumors, vector, and tumors treated with desARE3’UTR ERBB2-1, -3, and -30 with antibodies against ERBB2, CNOT1, UPF3B, and XRN1 (Supplementary Figures S14A–H). The results show that ERBB2 protein expression is significantly lost in mice that received the constructs compared to the controls (Supplementary Figure S14A, B). CNOT1 expression is significantly upregulated in desARE3’UTR ERBB2-3 (Supplementary Figure S14C, D), and UPF3B, XRN1, and caspase 7 are significantly upregulated in desARE3’UTR ERBB2-1, -3, and -30 compared to the controls (Supplementary Figures S14E–H; Figure 6F). This finding strongly validates our *in vitro* results across multiple cancers and validated the biology of design and the mechanistic of the engineered constructs.

## Discussion

To our knowledge, this is the first report of a demonstration of effective targeting of an oncogene by introducing destabilizing motifs into its 3’UTR resulting in destabilization of its transcript and degradation of its protein and consequent death of the cancer cells *in vitro* and inhibition of *in vivo* tumor growth. We have developed a novel technology by engineering the destabilized 3’UTR ARE that reliably degrades the oncogenic transcript and protein expression and their downstream kinases that cause cancer drug resistance. The findings provided in this study show that the deadly cancer drug resistance driven by pervasive oncogenic signals can, indeed, be controlled by this technology. This new technology demonstrates the ability to modulate levels of oncogenic transcripts by replacing the stabilizing with the destabilizing elements in the 3ʹ UTR in difficult-to-treat cancers. This work represents a technical advancement and a new strategy for reducing oncogene expression and their interactome. We provide proof-of-principle evidence in this study with findings from diverse cancer models and established a new paradigm which has opened the doors to applying this technique to control any transcript of interest in the temporal and spatial scale.

This technology is versatile and generally applicable to controlling diverse oncogenes and cellular transcripts across any tissue of interest. In this study, we show that we can control ERBB2 across a wide range of cancers, namely, NCI-H1975 ERBB2-expressing osimertinib-resistant EGFR T790M non-small-cell lung cancer, wildtype ERBB2+ breast cancer BT474, BT474 clone 5 ERBB2+ trastuzumab-resistant breast cancer, NCI-H2030 ERBB2-mutated non-small-cell lung cancer, and ERBB2-expressing HCT116 colorectal cancer. The technology is tissue-agnostic, but oncogene-targeted.

The breakthrough in the functionality of this novel method is the accuracy of biology by design based on universal gene regulatory principles. We designed the constructs to have the destabilized sequences of the ARE as described in the methods in which all the possible stabilizing elements were changed (Geisberg et al, 2014; Geissler et al 2016), and that this destabilized ARE will be driven by mRNA decapping protein DCP1A which will specifically upregulate the mRNA decay pathway and trigger the deadenylases CNOT1 and cleavage enzyme XRN1 to degrade the transcript of interest. Also integrated in the design is the sustained slow decay rate in the constructs. Some nucleotides were modified as described in Torabi et al (2021) to achieve a slow decay rate destabilizing construct. Our experimental results show that the optimal constructs worked as designed in destabilizing the ERBB2 transcript, degrading the transcript through mRNA deadenylases CNOT1 and XRN1, and by nonsense-mediated decay. The destabilizing constructs integrated into the genome and outcompeted the endogenous ERBB2 and degraded its transcript and protein.

The destabilization of the oncogene transcript we have described here is versatile. We have shown that the approach works in the oncogene of interest regardless of the tissue. We demonstrated that ERBB2 can be targeted and controlled with tumor death across many tissues and in diverse biological backgrounds, namely, in EGFR T790M lung cancer, in ERBB2+ wildtype breast cancer, in ERBB2+ trastuzumab-resistant breast, and in ERBB2-mutated lung cancer and ERBB2-expressing colorectal cancer. Our results suggest that this technology has the potential to be superior in controlling cancer chemoresistance driven by oncogene and its kinases. In osimertinib- and trastuzumab-resistant cancers, we show that once we control the oncogenes, we controlled the kinase signaling pathways and interactome through which they function to become resistant. For example, in ERBB2-overexpressing EGFR T790M lung cancer, we controlled and degraded ERBB2 and as well degraded EGFR, MET, and YES1 kinases that are known to drive this tumor resistance to drugs. The same finding was made in ERBB2+ trastuzumab-resistant breast cancer, in that the control of ERBB2 leads to loss of kinases such as WNK1 and YES1 implicated in this cancer mechanism of resistance.

The optimal constructs show the difference in the rate of degradation of ERBB2. This is explained by the method of cloning the constructs and the evolutionary pressure introduced in the destabilizing constructs by the *E. coli* in which they were cloned into. desARE3’UTR ERBB2-1, -2, -3, and -4 were obtained by cloning the constructs into recombinant-efficient *E. coli* ((Supplementary Figure S7). Of these four constructs, desARE3’UTR ERBB2-3 and desARE3’UTR ERBB2-1 degraded both the transcript and protein in most ERBB2 cellular settings both in wildtype and drug-resistant settings. desARE3’UTR ERBB2-2 downregulated the transcript and protein in the native ERBB2 WT setting but did not perform optimally in the ERBB2 drug-resistant setting. desARE3’UTR ERBB2-4 was the least performing. We extended our work by using Gibson assembly to clone the constructs in a recombinant-deficient *E. coli*. We obtained desARE3’UTR ERBB2-30 (Supplementary Figure S8). desARE3’UTR ERBB2-30 is effective as desARE3’UTR ERBB2-1 and -3 in degrading ERBB2 across multiple ERBB2 cancer types. desARE3’UTR ERBB2-30 has little to no mutation in the engineered destabilized ARE that we designed and marked in red underlined bars in (Supplementary Figure S4). deARE3’UTR ERBB2-1 and -3 contain some point mutation introduced by the recombinant efficient *E. coli*. Pointing to the fact that bacterial evolutionary pressure further selected and enhanced our construct’s function, it is worthwhile to mention that we designed and engineered the destabilizing constructs based on the BT474 wildtype ERBB2 3’UTR poly U mRNA-stabilizing elements. Yet, they work across various ERBB2-expressing cancers from diverse biological organs. The constructs showed anti-cancer activity such as impaired cancer cell viability, reduced migration and induction of apoptosis, and increased caspase 3/7, cleaved caspase 3, and caspase 9 expressions.

We found that our constructs do not kill normal breast epithelial cell MCF10A (Supplementary Figure S2H). These proof-of-principle findings strongly suggest that our destabilizing constructs do not affect normal cells but are cancer-specific and on target, which is very promising and only works in cancers carrying the stabilizing ERBB2 elements. We show that destabilizing ERBB2 does not affect c-MYC and its kinases (Figure 5A). Our results and finding are now validated in an animal tumor-bearing model, where the tumor volume is significantly reduced by the engineered destabilized constructs, and no abnormal vital organ functions or electrolyte imbalance were found (Figure 8; Supplementary Figures S12, S13). Technologies such as CRISPR (Zhang et al, 2015), RNAi (Fire et al., 1998), the degron system (Dohmen et al., 1994), and PROTAC (Sakamoto et al, 2001) are competitors to the findings presented in this study. CRISPR has been used to knockout gene expression with high efficiency of up to 90% loss of protein. However, CRISPR has remarkably high off-target effects and can cause unwanted chromosomal rearrangements as has been shown. Regarding RNAi, even though it can interfere and silence the expression of a protein, it also has off-target effects in order of magnitude greater than CRISPR. The degron system has been effective in mediating degradation of proteins; however, the system relies on inducibility by auxin or similar molecules to initiate its functions. The degron degradation is leaky and requires high levels of auxin (Yesbolatova et al 2020). Last, for PROTAC which targets proteins that have already been made, the limitation of PROTAC is highly toxic, costly to make, targets only intracellular proteins, and cannot target membrane proteins. This engineered destabilized 3’UTR-mediated degradation of the transcript offers a unique and novel approach to target oncogenic transcripts at their untranslated region which has been difficult to study. The engineered destabilized 3’UTR AREs are easy to synthesize as gBlocks, cheap and easy to clone, and they are very specific to target oncogenes in cancer cells but do not degrade proteins in normal cells, and the therapeutic degradation of transcript can be achieved at a very low dose of a nanogram of vectors containing the destabilizing constructs. This makes the technology very attractive. We have shown that in certain genetic settings of cancer as in ERBB2 HCT116, we are superior to CRISPR, RNAi, and shRNA in a head-to-head comparison. This is because these technologies show genetic dependencies, whereas our technology is agnostic to these dependencies and degrades the transcript once the stabilizing elements are destabilized (Table 1). We show that our constructs reprogram the ERBB2 genetic programs toward the normal individual ERBB2 gene program from diverse ethnicity (Figures 7A,B). By optical genome mapping, the *de novo* rare variants and genomic alterations are not different between the wildtype, vector, and ERBB2 mRNA-destabilized cancer (Figures 7C,D). This evidence strongly suggests that our constructs are safe for the cells. Infact, our data in Figure 7D show that desARE3’UTR ERBB2-3 also leads to loss of aneuploidy in cancer cells, and aneuploidy is a major cause of chromosomal instability, a key hall mark of cancer cells. Taken together, our data demonstrate that we can control ERBB2 across many cancer types and as well control the major drivers of cancers, which is aneuploidy without causing genome rearrangement. Future work will extend this technology to target more oncogenes across different disease settings and potentially any transcript of interest.

We have developed a novel technology by engineering the 3’UTR poly U-rich elements of ERBB2 from a stable to an unstable form. This approach destabilized and degraded ERBB2 and its kinases by overwriting the endogenous ERBB2 mRNA message across many cancer ERBB2 models and achieved primary tumor control *in vivo*. This technology is safe and caused no blood dyscrasia or vital organ damage or electrolyte changes. We found no evidence of genome rearrangement.

## Conclusion

We have developed a novel technology by engineering the 3’UTR AREs of ERBB2 from a stable to an unstable form. This approach destabilized and degraded ERBB2 and its kinases across many cancer ERBB2 models and achieved primary tumor control *in vivo*. This technology is safe and caused no blood dyscrasia or vital organ damage or electrolyte changes. We found no evidence of genome rearrangement.

## Data Availability

The original contributions presented in the study are publicly available. This data can be found here: https://zenodo.org/record/6968947, 10.5281/zenodo.6968947.

## Abbreviations

Cancer, ERBB2 (HER2), oncogene, drug-resistant cancer, EGFR T790M-driven non-small-cell lung cancer (NSCLC), trastuzumab-resistant breast cancer, colorectal cancer, therapeutic gene regulation, destabilized mRNA, 3’UTR genome engineering, tumor xenograft, mRNA message overwriting.
