# Supplementary material for: Destabilized 3’UTR elements therapeutically degrade ERBB2 mRNA in drug-resistant ERBB2+ cancer models
Source: Front Genet. 2023 Jun 9;14:1184600. doi: 10.3389/fgene.2023.1184600 (PMC10287955; doi:10.3389/fgene.2023.1184600)
Supplement: Supplementary file 1 [file DataSheet1.DOCX]

### Title

**Destabilized 3’UTR elements therapeutically degrade ERBB2 mRNA in drug resistant ERBB2+ cancer models**

### Authors

Chidiebere U Awah^1,^ ^2*^, Yana Glemaud^1^, Fayola Levine^1,2^, Kiseok Yang^1^, Afrin Ansary^1^, Fu Dong^1^, Leonard Ash^1^, Junfei Zhang^4^, Olorunseun O Ogunwobi^1,^ ^2*^

### Affiliations

1. Department of Biological Sciences, Hunter College of The City University of New York
2. Joan and Sanford I. Weill Department of Medicine, Weill Cornell Medicine, Cornell University, New York
3. Department of Pathology and Cell Biology & Department of System Biology, Columbia University Medical Center, New York

*Corresponding authors

Chidiebere U. Awah, MD, MSc, PhD and Olorunseun O. Ogunwobi, MD, PhD

Department of Biological Sciences, Hunter College of The City University of New York, New York, NY, USA

Joan and Sanford I. Weill Department of Medicine, Weill Cornell Medicine, Cornell University, New York, NY, USA

Email:

*[cha4010@med.cornell.edu,](mailto:cha4010@med.cornell.edu) [oo158@hunter.cuny.edu](mailto:oo158@hunter.cuny.edu)

**Supplementary Methods**

**QPCR with ERBB2 3’UTR primers**

To determine the stabilizing AU rich elements on the 3’UTR of ERBB2, we made cDNA from the total RNA of BT474, MCF7, T47D and MDA MB231 using the Qiagen reverse transcription kit (Catalog no: 205311). We performed RT-PCR using the cDNA according to the Qiagen manufacturer’s protocol using the following primer sequences in **Supplementary Table S1**:

PCR Cycle for cDNA synthesis:

94^o^C for 2mins

94^o^C for 15sec

55^o^C for 30sec

68-72 for 1min

Step2-3 for 40 cycles

4^o^C hold

PCR set up

38.1µl of H20

1.5µl of 50mM MgCl_2_

1µl of 10mM DNTP

1µl ERBB2 Forward primers

1µl ERBB2 Reverse primers

2µl of cDNA (200ng).

1µl of Taq polymerase

The amplicon was separated on 2% agarose gel.

2.8. Miniprep of vector

A stab of the vector was inoculated into LB media and grown over night shaking at 250rpm at 37^o^C. The plasmid vector gDNA was extracted using the Qiagen Midikit (Cat no: 12943).

Digest of vector with BstBI and BamH1

The vector was digested in BstBI and BamH1 at 37^o^C overnight using NEB buffer 2.1

1µl (400ng) of vector gDNA

1µl of Bstb1 and Bamh1 respectively

3µl of buffer

25µl of H20

Gel extraction of vector

The digested vector was gel extracted using Qiagen gel extraction kit (Cat no: 28704)

**Insertion of the gblock of the destabilized ERBB2 3’UTR into the digested vector.**

Digest insert with BstB1 and BamH1

The synthetic gblock containing the destabilized ERBB2 3’UTR ARE were digested with BstB1 and BamH1 in NEB buffer 2.1 for 1hr at 37^o^C

1µl (200ng) of gblock DNA

1µl of Bstb1 and Bamh1 respectively

3µl of buffer

25µl of H20

**Gel extraction of insert**

The digested synthetic gblock containing the destabilized ERBB2 3’UTR was gel extracted using Qiagen gel extraction kit (Cat no: 28704)

**Ligation**

2µl T4 ligase buffer

0.5µl Plasmid vector

4µl Insert (digested synthetic gblock)

20µl Nuclease free water

1µl T4 ligase (NEB)

At 22^o^C for 3hrs

**Transformation**

We transformed competent recombination efficient, E-coli (NEB 5 alpha, Cat no: C298H) using SOC media.

25µl of each cell was incubated on ice with 5µl of the ligation mix for 30mins. After 30mins was heat shocked in water bath at 42^o^C for 30sec. Tubes were placed back on ice for 2mins and 900µl of SOC media was added and then incubated at 37^o^C shaking for 1hr at 250rpm. After which they were plated on LB Agar plate containing ampicillin and the plate was incubated overnight at 37^o^C

**Colony picking and Miniprep**

Colonies were picked with pipette tips and inoculated into 5ml LB media containing Ampicillin and grown overnight at 37^o^C shaking at 250rpm. The pellets were spurned down and gDNA was extracted using the Qiagen Midikit (Cat no: 12943). And using a nanodrop machine the gDNA were quantified.

**Colony PCR with ERBB2 3’UTR primers and DCP1A promoter primers**

We performed PCR with ERBB2 primers and DCP1A primers using the PCR cycle described above.

Gel extraction

The colony PCR products of ERBB2 3’UTR and DCP1a promoters was gel extracted using Qiagen gel extraction kit (Cat no: 28704)

**Sanger sequencing**

We sequenced the cloned synthetic gblock amplicons of ERBB2 3’UTR using the primers described above by Sanger Sequencing with Psomagen Inc, Brooklyn New York. By cloning we obtained 4 clones that matched to the ERBB2 3’UTR: des (destabilized) ARE ERBB2-1, des ARE 3’UTR ERBB2-2, des ARE 3’UTR ERBB2-3, des ARE 3’UTR ERBB2-4 (**Supplementary Figure S7**)

**Design of Gibson Assembly Primers**

**Gibson Assembly**

We succeeded in cloning the destabilized ERBB2 3’UTR into the vector by ligation, however, to extend this approach, we used GIBSON Assembly and succeeded in cloning ERBB2 destabilized 3’UTR synthetic constructs into the plasmid vector.

**ERBB2 and Vector Gibson Assembly primers design**

We loaded the sequences of the synthetic gblock of destabilized ARE 3’UTR ERBB2 and vector (Sp6 vector) into the NEB builder (<https://nebuilder.neb.com/#!/>) which generated the following primers to amplify the ERBB2 and Sp6 vector primers for Gibson Assembly , see **Supplementary Table** **S1**.

**Gibson Assembly of the destabilized 3’UTR of ERBB2**

PCR amplification Vector (Sp6) and Insert (destabilized 3’UTR ERBB2).

6µl H20

1µl of ERBB2/Sp6 vector Forward primers respectively into a tube

1µl of ERBB2/Sp6 vector Reverse primers respectively into a tube

2µl of gDNA (200ng).

10µl of Q5 high fidelity polymerase 2X master mix (NEB cat: M0492S).

Gibson Assembly PCR cycle

98^o^C for 30sec

98^o^C for 10sec

55^o^C for 30sec

68-72 for 1min

Step2-3 for 38 cycles

4^o^C hold

**NEB HIFI DNA Assembly**

0.5µl of Sp6 vector mixed with 4µl of insert (destabilized 3’UTR ERBB2).

10µl of NEB HiFi DNA assembly master mix (NEB cat no: E2621S).

5.5µl of deionized water

Placed at 50^o^C for 15mins.

**Transformation Recombination deficient E. coli (NEB Cat no C3019H).**

We transformed deficient competent E-coli with the DNA assembly using SOC media

25µl of each cell was incubated on ice with 5µl of the ligation mix for 30mins. After 30mins was heat shocked in water bath at 42^o^C for 30sec. Tubes were placed back on ice for 2mins and 900ul of SOC media was added and then incubated at 37^o^C shaking for 1hr at 250rpm. After which they were plated on LB Agar plate containing ampicillin and the plate was incubated overnight at 37^o^C

Colony picking and Miniprep

Colonies were picked with pipette tips and inoculated into 5ml LB media containing Ampicillin and grown overnight at 37^o^C shaking at 250rpm. The pellets were spurned down and gDNA was extracted using the Qiagen Midikit (Cat no: 12943). And using a nanodrop machine the gDNA were quantified.

Colony PCR with ERBB2 Gibson primers

We performed PCR with ERBB2 Gibson primers by using the PCR cycle described above.

Gel extraction

The colony PCR products of ERBB2 3’UTR Gibson assembled was gel extracted using Qiagen gel extraction kit (Cat no: 28704)

Sanger sequencing of the cloned destabilized 3’UTR of ERBB2 amplicon

We sequenced colony PCR product of Gibson assembled ERBB2 3’UTR using the Gibson primers described above by Sanger Sequencing with Psomagen Inc, Brooklyn New York. We succeed in a obtaining a clone desARE3’UTR ERBB2-30 (**Supplementary Figure S8**).

Sequence alignment of wildtype cDNA, RNA versus the destabilized 3’UTR ERBB2 cDNA and RNA sequences

To confirm the engineered changes of the destabilized 3’UTR of ERBB2 versus the wildtype ERBB2 cDNA and RNA , we aligned the wildtype and the engineered destabilized and cloned sequences both on the RNA level with their control wildtype using the software Clustalw Omega (<https://www.ebi.ac.uk/Tools/msa/clustalo/>) (**Supplementary Figure S4**).

###
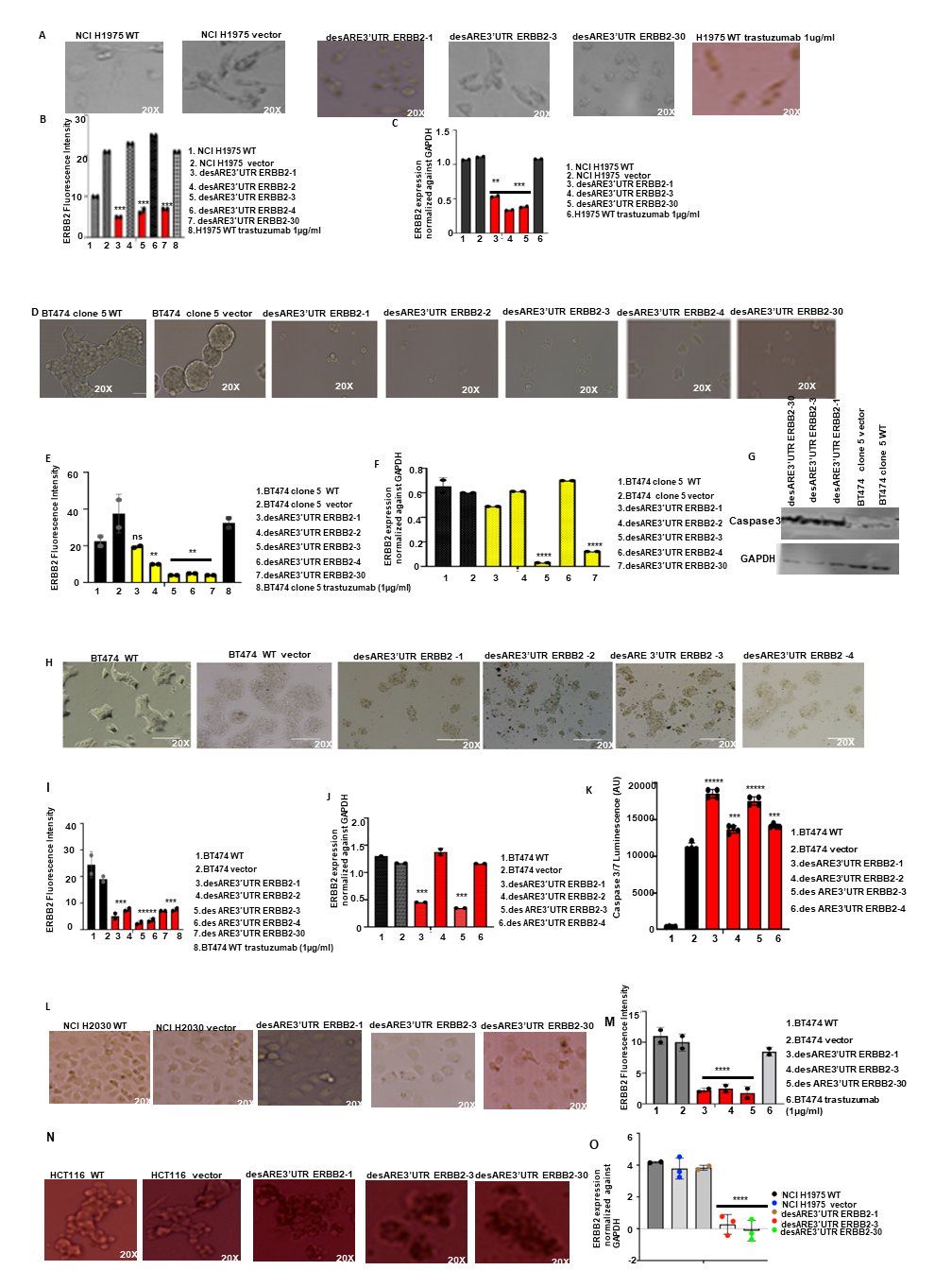


### Supplementary Figure 1. The destabilized 3’UTR of ERBB2 severely distorted lung cancer and breast cancer cells membrane, degraded ERBB2 transcript and lead to cell death.

1. Pictures show bright field microscope images of the NCI-H1975 wildtype cells, vector, cells containing constructs desARE3’UTR ERBB2-1, 3 and 30 and wild type trastuzumab treated NCI-H1975 cells (n=2).
2. Bar charts show quantification of the ERBB2 fluorescence expression on NCI- H1975 wildtype cells, vector and cells containing constructs desARE3’UTR ERBB2-1, 3, 30 (red bars), desARE3’UTR ERBB2-2,4 and wild type trastuzumab treated NCI-H1975 cells (n=2). T-test *** =0.003, WT , vector vs desARE3’UTRERBB2-1,3,30.
3. Bar charts show quantification of the ERBB2 western blot expression normalized against GAPDH on NCI-H1975 wildtype cells, vector and cells containing constructs desARE3’UTR ERBB2-1, 3, 30 (red bars) and wild type trastuzumab treated NCI-H1975 cells (n=2). T-test *** =0.0021 WT, vector vs desARE3’UTRERBB2-3,30, ** =0.02 desARE3’UTRERBB2-1
4. Pictures show bright field microscope images of the BT474 clone 5 wildtype cells, vector, cells containing constructs desARE3’UTR ERBB2-1,2, 3, 4 and 30 (n=3).
5. Bar charts show quantification of the ERBB2 fluorescence expression on BT474 clone 5 wildtype cells, vector and cells containing constructs desARE3’UTR ERBB2-1,2, 3, 4, 30 and trastuzumab treated cells (yellow bars) (n=2). T-test, ns= WT, vector vs desARE3’UTRERBB2-1,

**=0.037 WT, vector vs desARE3’UTRERBB2-2, **=0.0178 WT, vector vs desARE3’UTRERBB2-3,

**=0.0198 WT, vector vs desARE3’UTRERBB2-4, ** =0.0178 WT, vector vs desARE3’UTRERBB2-30.

1. Bar charts show quantification of the ERBB2 western blot expression normalized against GAPDH on BT474 clone 5 wildtype cells, vector and cells containing constructs desARE3’UTR ERBB2-1, 2, 3, 4 and 30 (yellow bars) (n=3). T-test ****=0.00012 WT, vector vs desARE3’UTRERBB2-3,30.
2. Western blots show active caspase 3 and GAPDH expression across the wildtype BT474 clone 5, vector and on BT474 clone 5 containing constructs desARE3’UTR ERBB2-1, 3, and 30 (n=2).
3. Pictures show bright field microscope images of the BT474 wildtype cells, vector, cells containing constructs desARE3’UTR ERBB2-1,2, 3, and 4 (n=3).
4. Bar charts show quantification of the ERBB2 fluorescence expression on BT474 wildtype cells, vector and cells containing constructs desARE3’UTR ERBB2-1,2, 3, 4 (red bars) (n=2). T -test, **=0.0331 WT, vector vs desARE3’UTRERBB2- 1, **=0.0040 WT, vector vs desARE3’UTRERBB2-2,**=0.0249 desARE3’UTRERBB2-3,

**=0.0272 WT, vector vs desARE3’UTRERBB2-4, **=0.0377 WT, vector vs

desARE3’UTRERBB2-30, **=0.0406 BT474 trastuzumab treated cells.

1. Bar charts show quantification of the ERBB2 western blot expression normalized against GAPDH on BT474 wildtype cells, vector and cells containing constructs desARE3’UTR ERBB2-1, 2, 3, and 4 (red bars) (n=2). T- test, ***=0.0036 desARE3’UTRERBB2-1 and 3.
2. Bar charts show active caspase 3/7 luminescence on BT474 wildtype cells, vector and cells containing constructs desARE3’UTR ERBB2-1, 2, 3 and 4 (n=2). T-test,

*****<0.00001 WT, vector vs desARE3’UTRERBB2-1,3. ***=0.0022 WT, vector vs desARE3’UTRERBB2-2,4.

1. Pictures show bright field microscope images of the NCI-H2030 wildtype cells, vector, cells containing constructs desARE3’UTR ERBB2-1, 3 and 30 (n=2).
2. Bar charts show quantification of the ERBB2 fluorescence intensity on NCI H2030 wildtype cells, vector and cells containing constructs desARE3’UTR ERBB2-1, 3, 30 and trastuzumab treated cells (n=2). T-test, ****=0.0004 WT, vector vs desARE3’UTRERBB2-1,3,30.
3. Pictures show bright field microscope images of the HCT116 wildtype cells, vector, cells containing constructs desARE3’UTR ERBB2-1, 3 and 30 (n=2).
4. Bar charts show quantification of the ERBB2 expression normalized against GAPDH by qPCR on HCT116 wildtype cells, vector and HCT116 cells containing constructs desARE3’UTR ERBB2-1, 3 and 30 (n=2). T-test WT, vector vs (****=0.0003, desARE3’UTRERBB2-3

and 30).

###
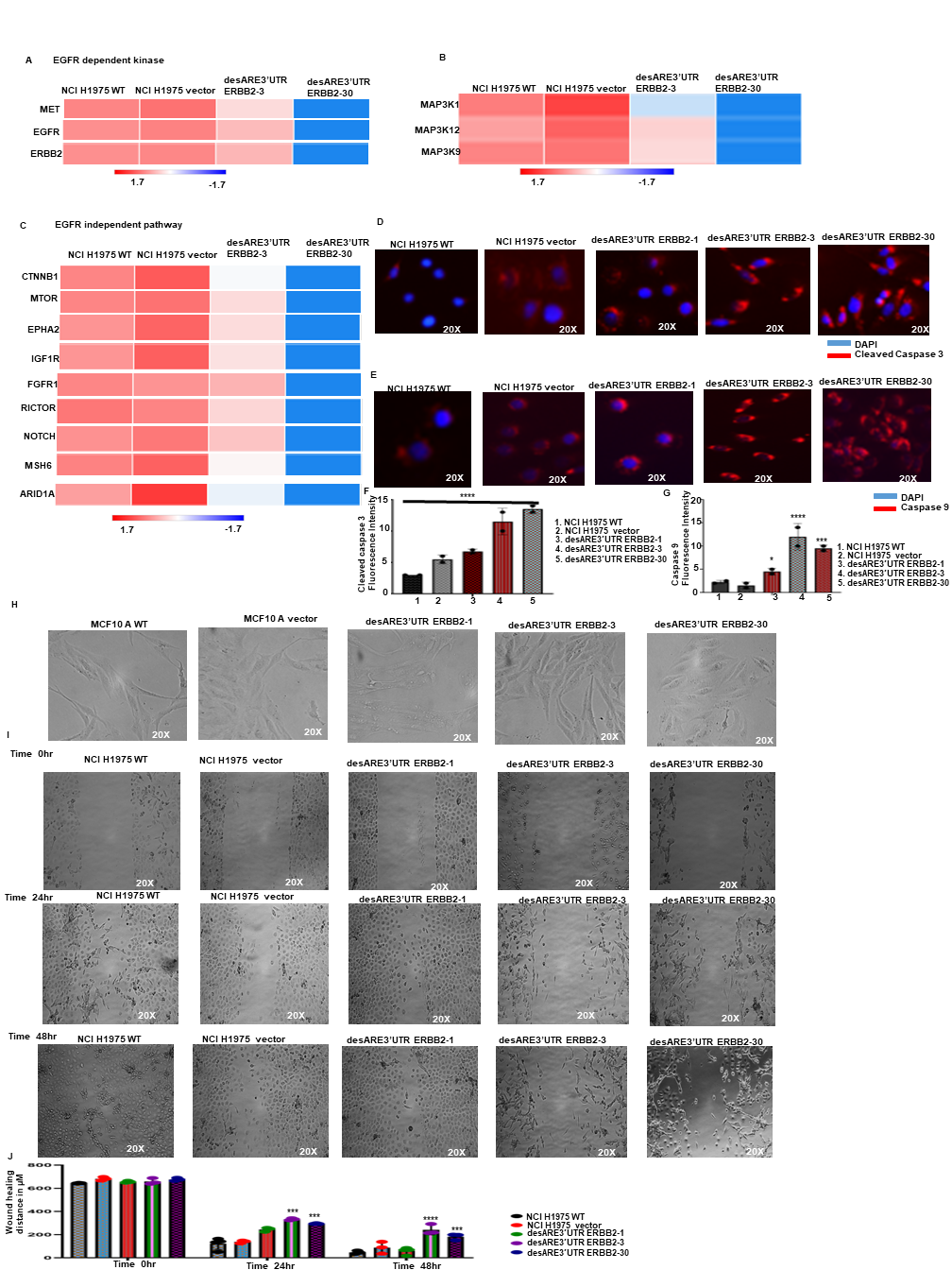


### Supplementary Figure 2. Engineered destabilized 3’UTR of ERBB2 degradation of ERBB2 in EGFR T90M reduces the expression of EGFR dependent and independent kinases in non-small cell lung cancer.

1. Heat map shows EGFR dependent kinases known to cause osimertinib resistance: EGFR, MET and ERBB2 expression changes in the wildtype NCI H1975, vector, desARE3’UTR ERBB2-3 and 30.
2. Heat map shows EGFR dependent kinases known to cause osimertinib resistance: MAP3K1, MAP3K12, MAP3K9 expression changes in the wildtype NCI H1975, vector, desARE3’UTR ERBB2-3 and 30.
3. Heat map shows EGFR independent genes known to cause osimertinib drug resistance: CTNNB1, MTOR, EPHA2, IGFR1, FGFR1, RICTOR, NOTCH, MSH6, ARID1A expression changes in the wildtype NCI H1975, vector, desARE3’UTR ERBB2-3 and 30.

## Immunofluorescence images show cleaved caspase 3 expression (stained in red) and nuclei (DAPI-blue) on wildtype NCI H1975, vector and on NCI H1975 containing constructs desARE3’UTR ERBB2-1, 3, 30 (n=2) Magnification (20x).

1. Immunofluorescence images show caspase 9 expression (stained in red) and nuclei (DAPI-blue) on wildtype NCI H1975, vector and on NCI H1975 containing constructs desARE3’UTR ERBB2-1, 3, 30 (n=2) Magnification(20x).
2. Bar charts show quantification of the cleaved caspase 3 fluorescence intensity on NCI H1975 wildtype cells, vector and cells containing constructs desARE3’UTR ERBB2-1, 3, 30 (n=2). T-test, ****=0.00025 WT, vector vs desARE3’UTRERBB2-3,30
3. Bar charts show quantification of the caspase 9 fluorescence intensity on NCI H1975 wildtype cells, vector and cells containing constructs desARE3’UTR ERBB2-1, 3, 30 (n=2). T-test, * p=0.05 WT, vector vs desARE3’UTR ERBB2-1,

****p=0.00025 WT, vector vs desARE3’UTRERBB2-3,30

***p=0.0022 WT, vector vs desARE3’UTR ERBB2-30.

1. Bright field microscope images of the MCF10A wildtype cells, vector, cells containing constructs desARE3’UTR ERBB2-1, 3 and 30. Magnification (20x).
2. Bright field microscope images of wound scratch and closing in wild type NCI H1975, vector, desARE3’UTR ERBB2-1,3,30 treated cells at 0hr, 22hr and 48hr. Magnification (20x).
3. Bar chart shows the quantification of wound opening at 0hr, 22 and 48hrs in NCI H1975 wildtype, vector, desARE3’UTR1,3,30. At 22hr WT, vector vs desARE3’UTRERBB2-3,30 show significant wound opening t-test ***p=0.0014 and at 48hr t-test **** p=0.0004 WT, vector vs desARE3’UTRERBB2-3, ***p=0.003 WT, vector vs desARE3’UTRERBB2-30. All experiment n=2.

###
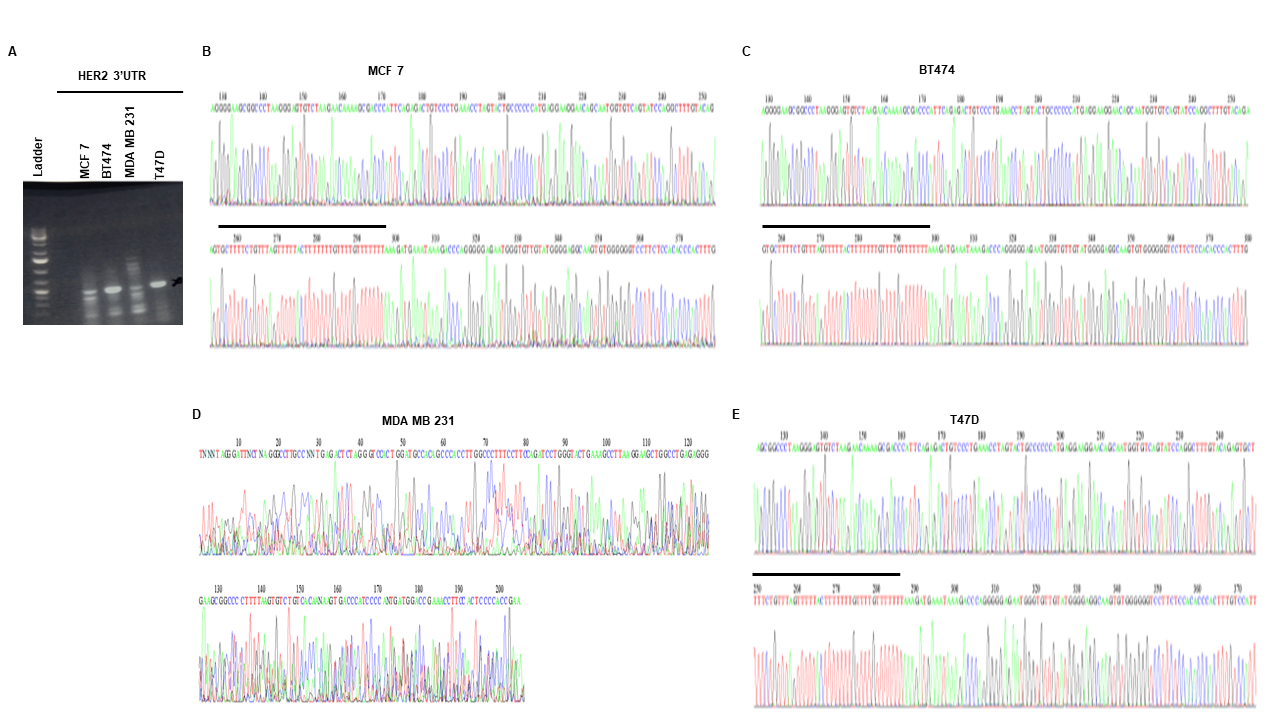


### Supplementary Figure 3: Identification of poly U stabilizing sequences on the 3’UTR of ERBB2

1. Gel picture shows the cDNA amplification of ERBB2 3’UTR across different cancers band size is 450bp marked in black asterisks.
2. Sanger sequencing electropherogram of ERBB2 3’UTR cDNA. The poly U stabilizing sequences are marked in black line in MCF7.
3. Sanger sequencing electropherogram of ERBB2 3’UTR cDNA. The poly U stabilizing sequences are marked in black line in BT474.
4. Sanger sequencing electropherogram of ERBB2 3’UTR cDNA. The poly U stabilizing sequences are not present in the triple negative breast cancer MDAMB231
5. Sanger sequencing electropherogram of ERBB2 3’UTR cDNA. The poly U stabilizing sequences are marked in black line in T47D.

###
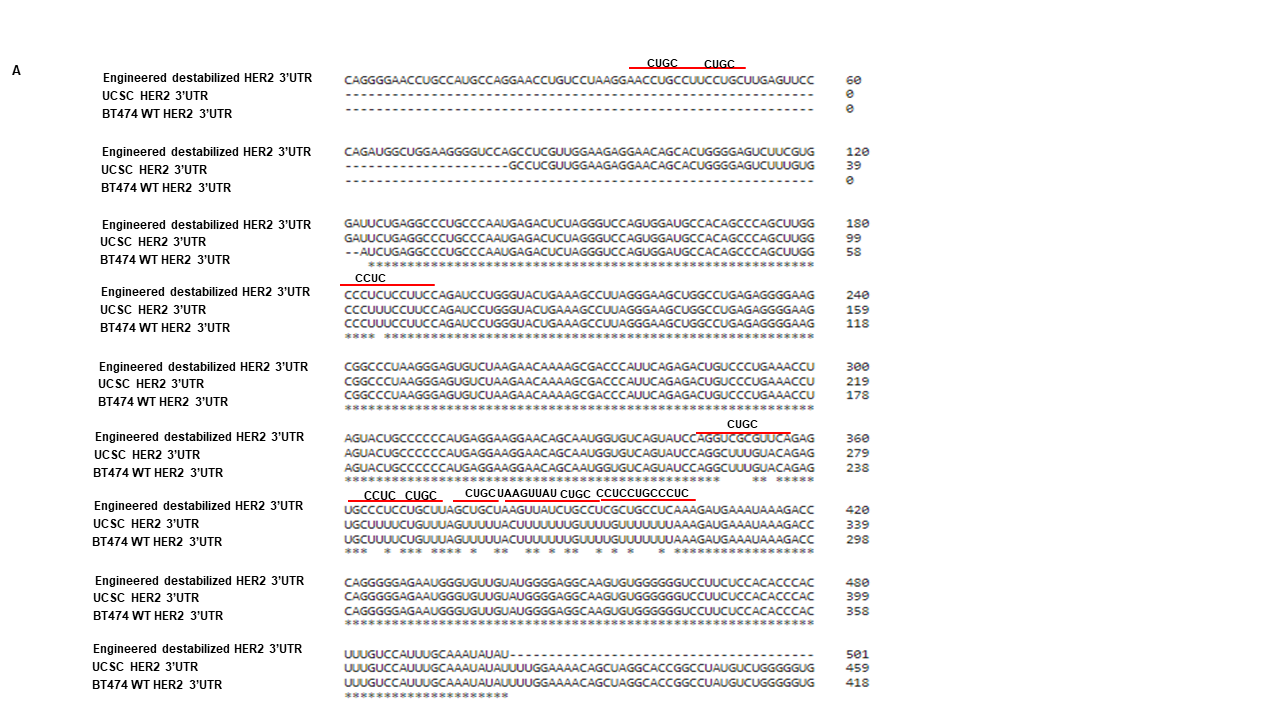


### Supplementary Figure 4. ERBB2 3’UTR cDNA converted to mRNA sequences with stabilizing poly U engineered to destabilize elements underlined in red.

**A.** Sequence alignment of the HER 3’UTR sequence from the UCSC genome browser, ERBB2 3’UTR sequence from the BT474 WT and the engineered ERBB2 3’UTR sequence. The sequence underlined in red line are the motif we replaced the stabilizing elements with.


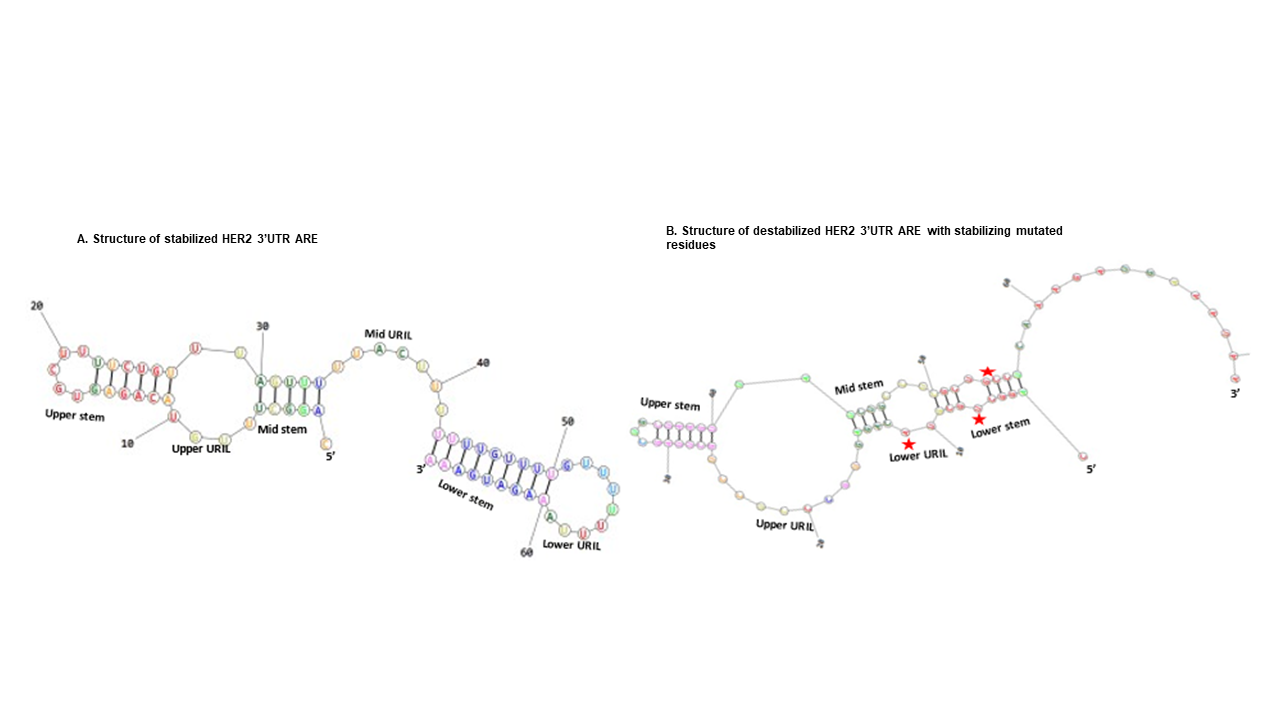


### Supplementary Figure 5. Structure model of stable ERBB2 3’UTR and destabilized ERBB2 3’UTR motif with increased stability due to mutated residues marked in red

**A-B.** Depiction of component of the stabilized and destabilized 3’UTR , asterisk in **B.** represents residues that will mutated to enhance the stability of destabilized 3’UTR elements.

###
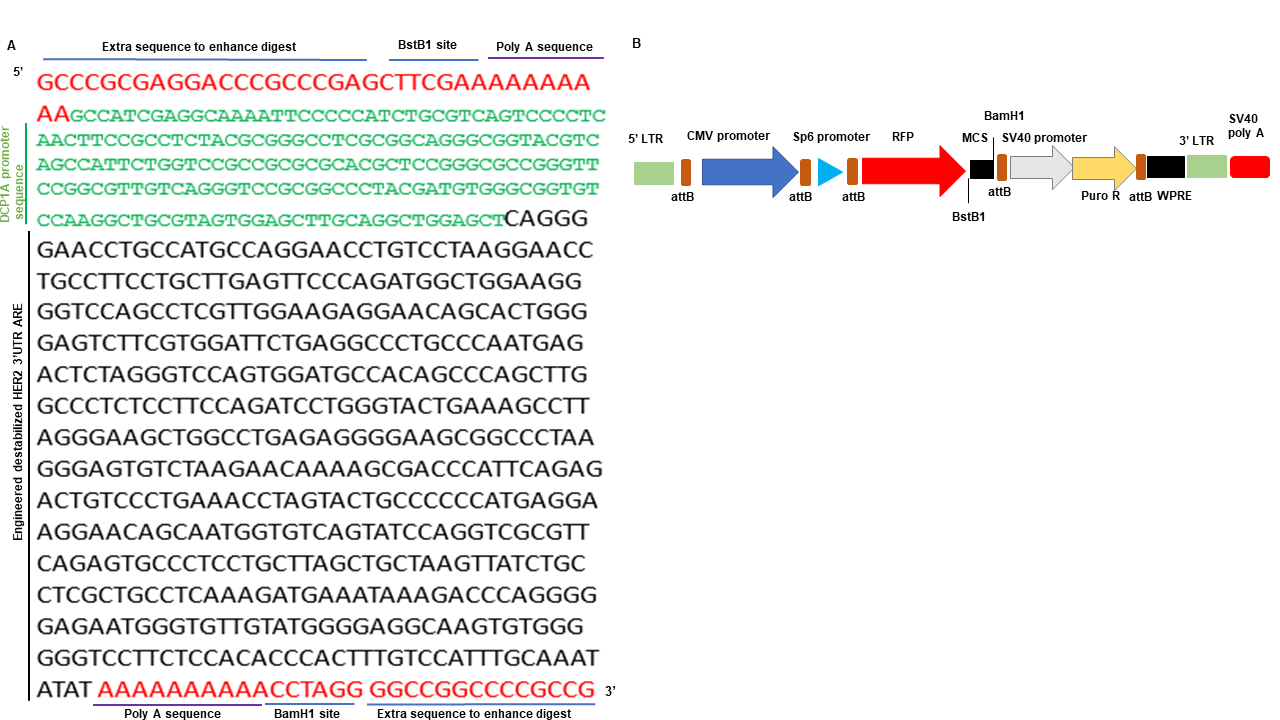


### Supplementary Figure 6. Design of destabilized ERBB2 3’UTR with DCP1A promoter, BstB1, BamH1 restriction sites and plasmid vector design

1. Schematic depiction of the design of the synthetic gblock showing nucleotides to enhance restriction digest, BstB1 sites, Poly A sequence, hDCP1A promoter (green), the engineered destabilized ERBB2 3’UTR ARE (black) and BamH1 sites.
2. Representation of the structure of the vector depicting the components parts and the restriction enzyme sites used at the multiple cloning site.


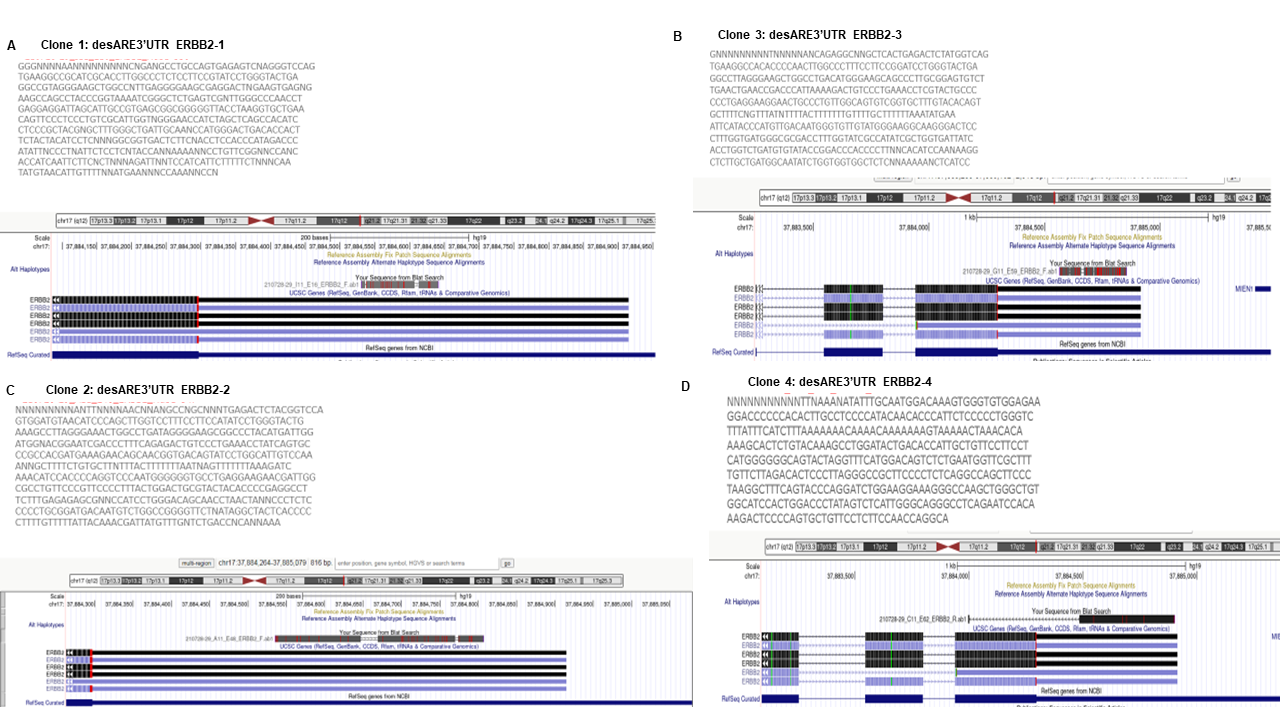


### Supplementary Figure 7. Identification of successfully engineered destabilized ERBB2 3’UTR ARE obtained by cloning in recombinant efficient E. coli.

**A-D.** Sequences of desARE3’UTR ERBB2-1, 2,3 and 4 clones mapped to the ERBB2 3’UTR

###
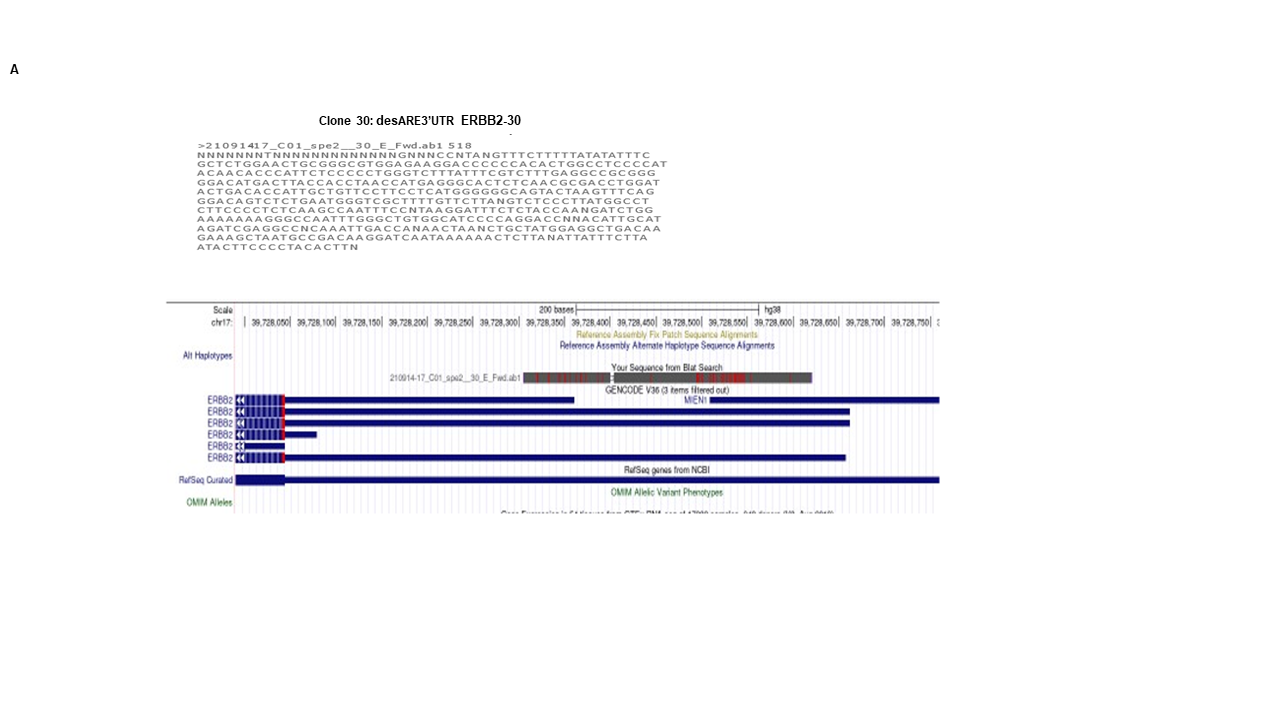


### Supplementary Figure 8. Identification of successful engineered destabilized ERBB2 3’UTR obtained by Gibson assembly cloned in recombinant deficient E. coli.

**A.** Sequence of desARE3’UTR ERBB2-30 clone mapped to the ERBB2 3’UTR.

###
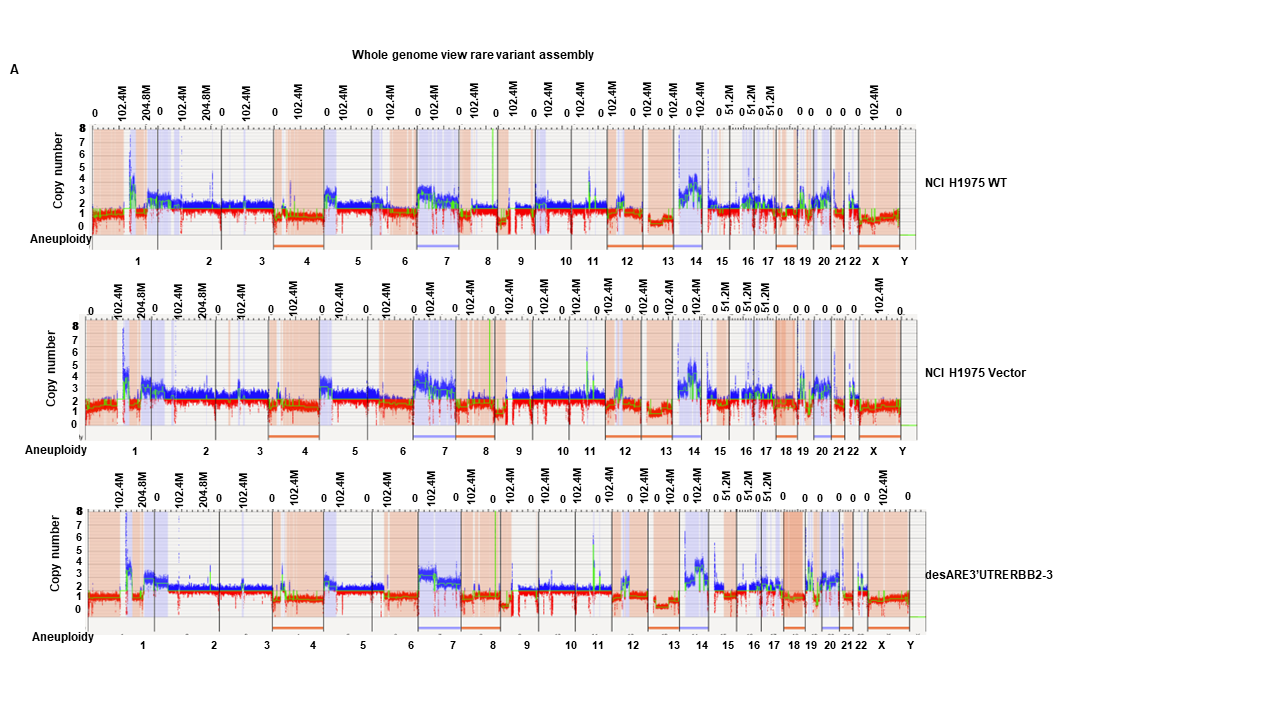


### Supplementary Figure 9: Whole genome view of rare variant assembly comparing wild type EGFR T790M, vector and desARE3’UTR ERBB2-3 non- small cell lung cancer cells.

**A.** Genome view of whole rare variant assembly in wild type NCI H1975, vector and desARE3’UTRERBB2-3 n=2


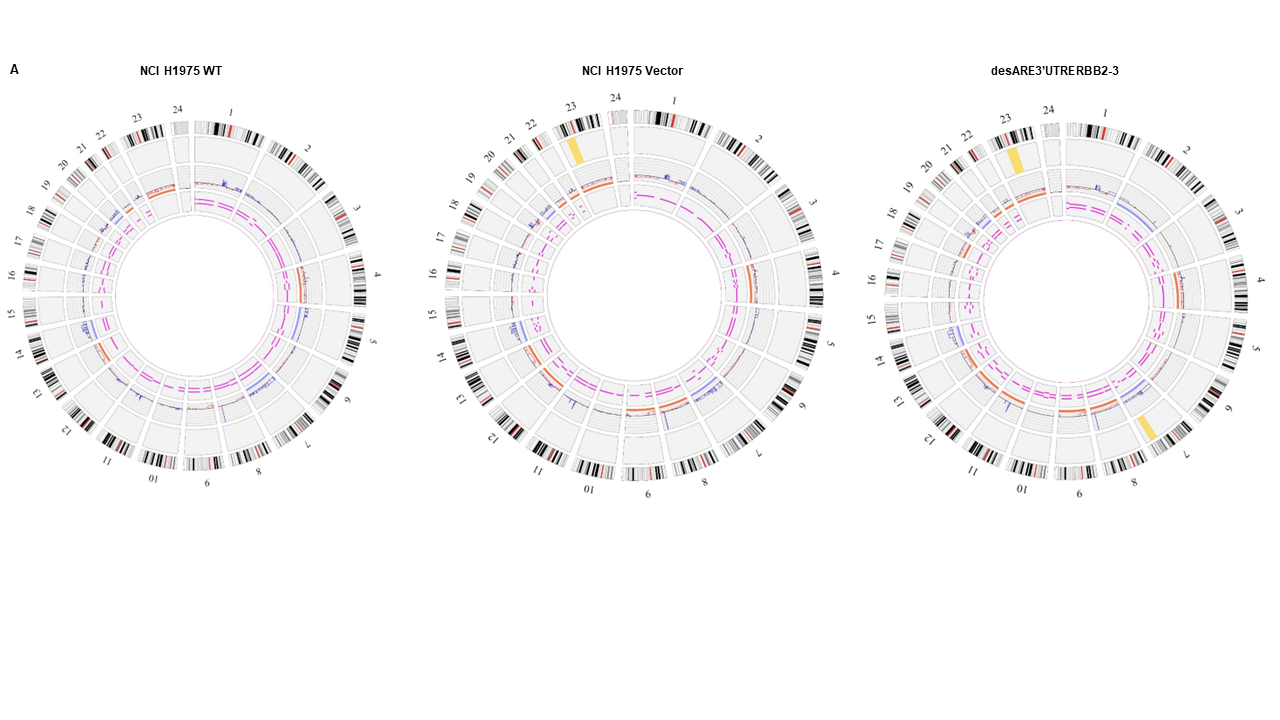


### Supplementary Figure 10. Whole genome view of rare variant assembly comparing wild type EGFR T790M, vector and desARE3’UTR ERBB2-3 non- small cell lung cancer cells.

**A.** Circos plot of de novo assembly in wild type NCI H1975, vector and desARE3’UTRERBB2-3. n=2

###
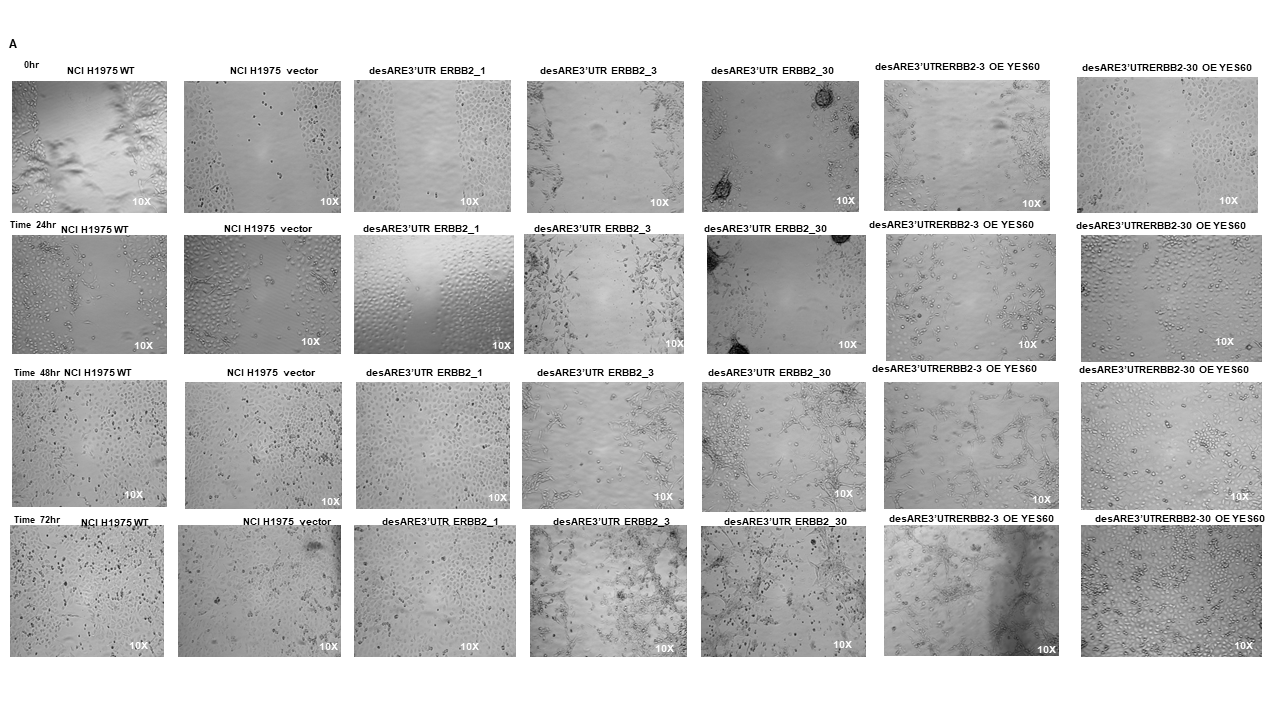


### Supplementary Figure 11. Migration assay of NCI H1975 WT, vector, desARE 3’UTRERBB2-1, 3, 30 and destabilized cells with over expressed YES1.

**A.** Microscope images of wound healing assay of NCI H1975 WT, vector, desARE3’UTRERBB2-1, 3 and 30 and desARE3’UTRERBB2-3 and 30 overexpressed with YES1.Images were taken at 0hr, 24hr, 48hrs and 72hrs at 10x.

#
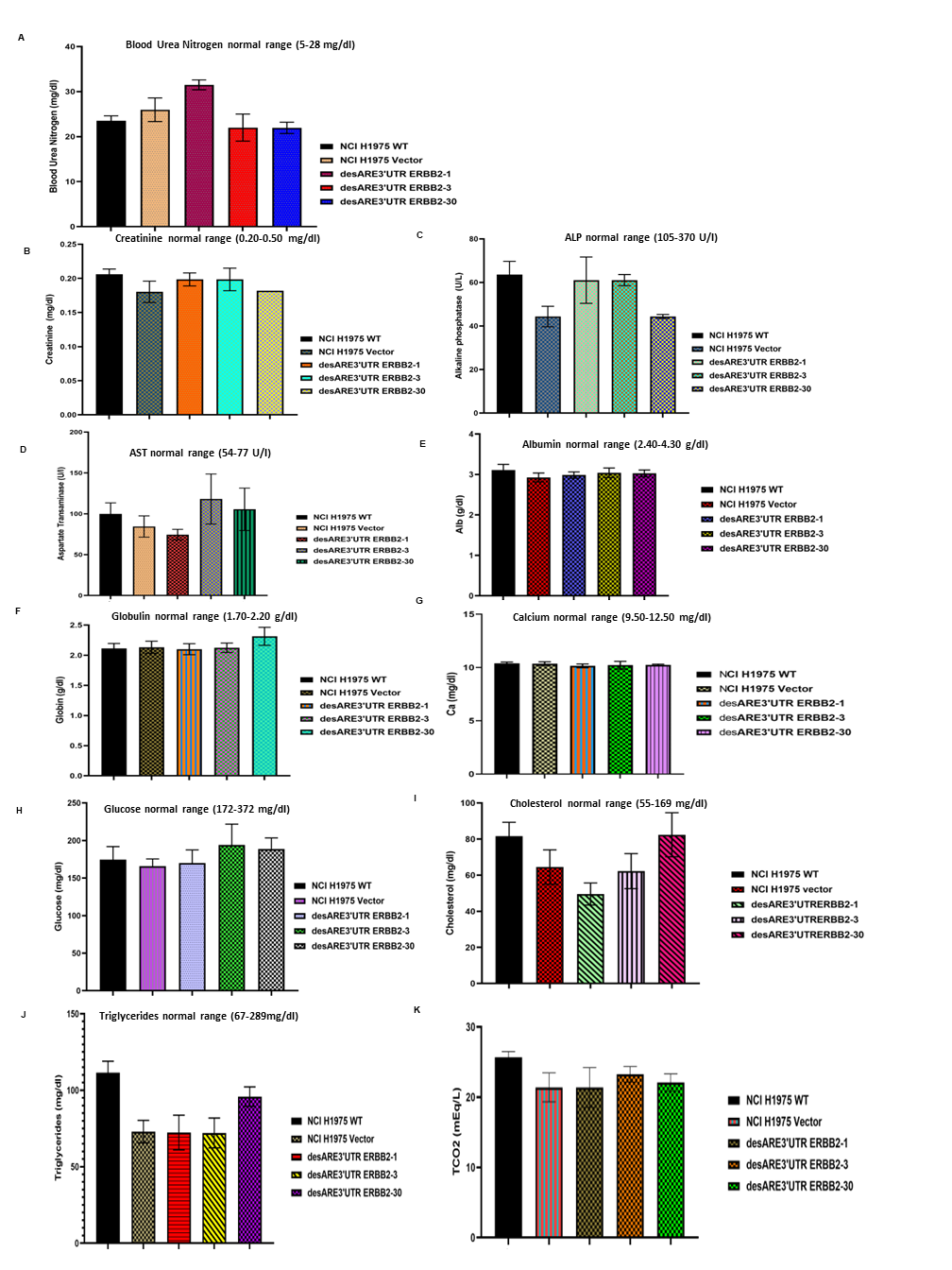


# Supplementary Figure 12. Engineered destabilized 3’UTR of ERBB2 does not affect the vital organ function not normal cell electrolyte balance.

1. Bar charts show blood urea nitrogen level of mice bearing tumor NCI H1975 WT (black), vector (yellow), and mice treated with desARE3’UTRERBB2- 1(light green), desARE3’UTRERBB2-3 (green) and desARE3’UTRERBB2- 30 (red).
2. Bar charts show creatinine levels of mice bearing tumor NCI H1975 WT (black), vector (gray), and mice treated with desARE3’UTRERBB2-1(orange), desARE3’UTRERBB2-3 (light green) and desARE3’UTRERBB2-30 (brown).
3. Bar charts show alkaline phosphatase level of mice bearing tumor NCI H1975 WT (black), vector (blue), and mice treated with desARE3’UTRERBB2-1(light green), desARE3’UTRERBB2-3 (dark green) and desARE3’UTRERBB2-30 (white on black crosses).
4. Bar charts show aspartate amino transferase of mice bearing tumor NCI H1975 WT (black), vector (orange), and mice treated with desARE3’UTRERBB2-1(red), desARE3’UTRERBB2-3 (white on black crosses) and desARE3’UTRERBB2-30 (green with black lines).
5. Bar charts show albumin level of mice bearing tumor NCI H1975 WT (black), vector (red), and mice treated with desARE3’UTRERBB2-1(blue), desARE3’UTRERBB2-3 (orange) and desARE3’UTRERBB2-30 (purple).
6. Bar charts show globulin of mice bearing tumor NCI H1975 WT (black), vector (yellow stripped), and mice treated with desARE3’UTRERBB2-1(orange), desARE3’UTRERBB2-3 (white shaded) and desARE3’UTRERBB2-30 (light green).
7. Bar charts show calcium level of mice bearing tumor NCI H1975 WT (black), vector (yellow stripped), and mice treated with desARE3’UTRERBB2-1(blue stripped), desARE3’UTRERBB2-3 (green stripped) and desARE3’UTRERBB2-30 (pink stripped).
8. Bar charts show glucose level of mice bearing tumor NCI H1975 WT (black), vector (pink), and mice treated with desARE3’UTRERBB2-1(light blue), desARE3’UTRERBB2-3 (green stripped) and desARE3’UTRERBB2-30 (black stripes on white).
9. Bar charts show cholesterol level of mice bearing tumor NCI H1975 WT (black), vector (red), and mice treated with desARE3’UTRERBB2-1(green stripe), desARE3’UTRERBB2-3 (light pink) and desARE3’UTRERBB2-30 (purple stripe).
10. Bar charts show triglyceride level of mice bearing tumor NCI H1975 WT (black), vector (light yellow), and mice treated with desARE3’UTRERBB2- 1(red stripes), desARE3’UTRERBB2-3 (bright yellow stripes) and desARE3’UTRERBB2-30 (purple stripes).
11. Bar charts show blood level of C02 of mice bearing tumor NCI H1975 WT (black), vector (red stripes), and mice treated with desARE3’UTRERBB2- 1(orange stripes), desARE3’UTRERBB2-3 (yellow stripes) and desARE3’UTRERBB2-30 (green stripes)

###
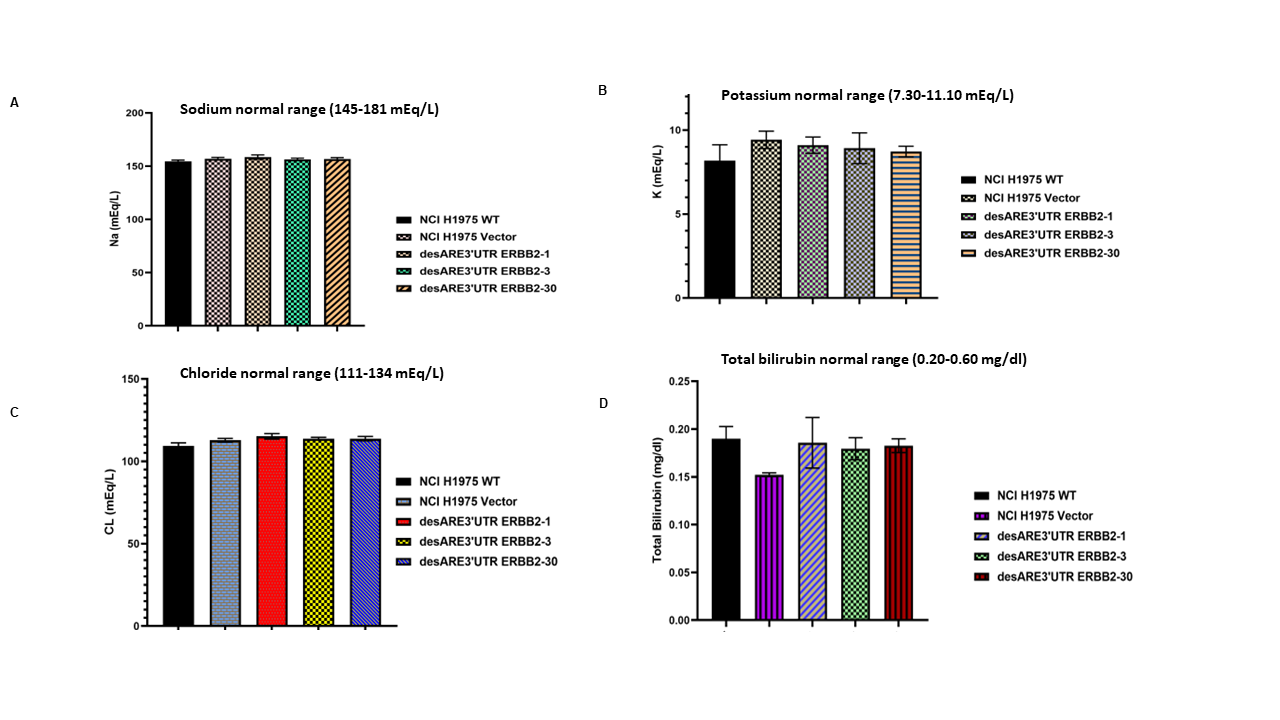


### Supplementary Figure 13. Analysis of electrolyte levels in NCI H1975 WT, vector in mice bearing tumors and in mice bearing tumors treated with desARE3’UTR ERBB2-1,3 and 30.

1. Bar charts show blood sodium level of mice bearing tumor NCI H1975 WT (black), vector (pink), and mice treated with desARE3’UTRERBB2-1(light blue stripes), desARE3’UTRERBB2-3 (green stripes) and desARE3’UTRERBB2- 30 (yellow stripes).
2. Bar charts show blood potassium level of mice bearing tumor NCI H1975 WT (black), vector (yellow stripes), and mice treated with desARE3’UTRERBB2- 1(green stripes), desARE3’UTRERBB2-3 (blue stripes) and desARE3’UTRERBB2-30 (yellow stripes).
3. Bar charts show blood chloride of mice bearing tumor NCI H1975 WT (black), vector (blue), and mice treated with desARE3’UTRERBB2-1(red), desARE3’UTRERBB2-3 (yellow) and desARE3’UTRERBB2-30 (blue stripes).
4. Bar charts show total bilirubin levels of mice bearing tumor NCI H1975 WT (black), vector (pink), and mice treated with desARE3’UTRERBB2-1(blue), desARE3’UTRERBB2-3 (green stripes) and desARE3’UTRERBB2-30 (red stripes).


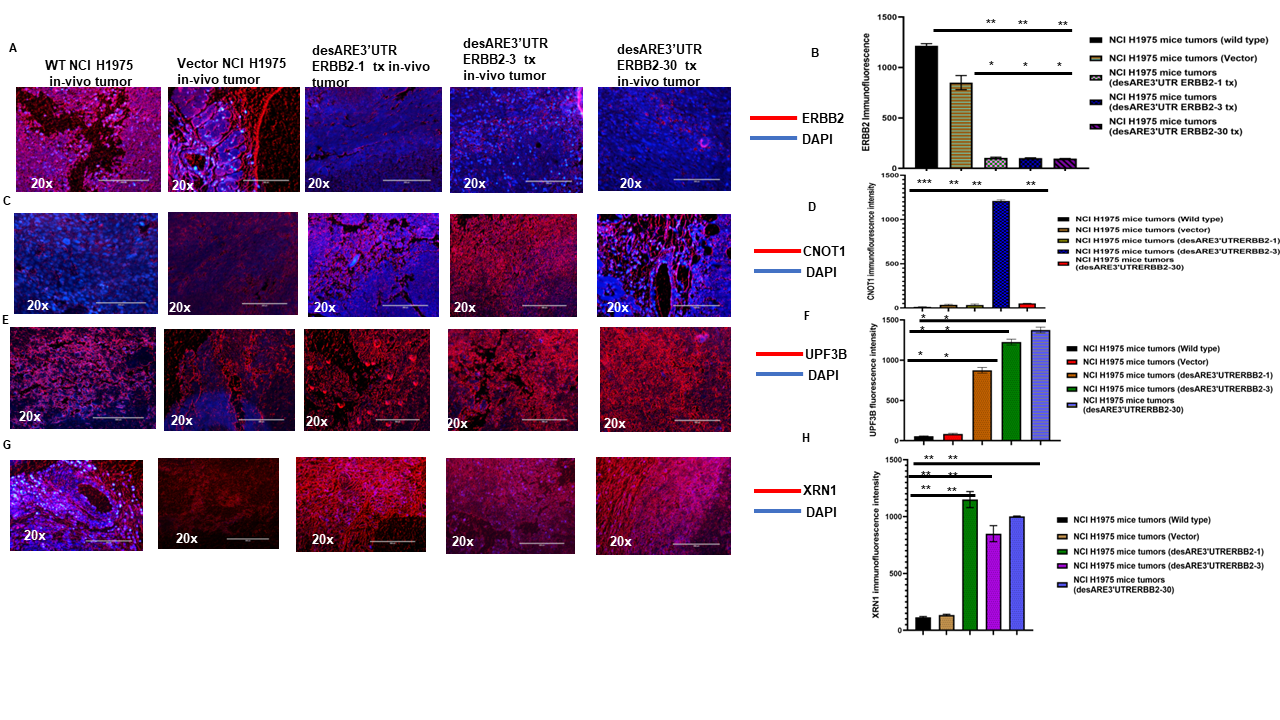
**Supplementary Figure 14**. Immunofluorescence staining of xenografts tumors for ERBB2, CNOT1, UPF3B and XRN1.

1. Immunofluorescence pictures show ERBB2 expression (stained in red) and nuclei (DAPI-blue) on xenograft tumors, wildtype NCI-H1975, vector and on tumors treated with constructs desARE3’UTR ERBB2-1, 3 and 30. Magnification (20x).
2. Bar chart show quantification of ERBB2 protein fluorescence intensity on xenograft tumors, wildtype NCI-H1975, vector and on tumors treated with constructs desARE3’UTR ERBB2-1, 3 and 30. T-test of WT vs desARE3’UTR ERBB2-1,3,30 (p=0.0074, 0.0057, 0.0094). T-test on Vector vs desARE3’UTRERBB2-1,3,30 (p=0.038, 0.0409, 0.0434)
3. Immunofluorescence pictures show CNOT1 expression (stained in red) and nuclei (DAPI-blue) on xenograft tumors, wildtype NCI-H1975, vector and on tumors treated with constructs desARE3’UTR ERBB2-1, 3 and 30. Magnification (20x).
4. Bar chart show quantification of CNOT1 protein fluorescence intensity on xenograft tumors, wildtype NCI-H1975, vector and on tumors treated with constructs desARE3’UTR ERBB2-1, 3 and 30. T- T-test of desARE3’UTR ERBB2-3 vs WT, vector, desARE3’UTR ERBB2-1,30 (p=0.0040, 0.0008, 0.0027, 0.0163).
5. Immunofluorescence pictures show UPF3B expression (stained in red) and nuclei (DAPI-blue) on xenograft tumors, wildtype NCI-H1975, vector and on tumors treated with constructs desARE3’UTR ERBB2-1, 3 and 30. Magnification (20x).
6. Bar chart show quantification of UPF3B protein fluorescence intensity on xenograft tumors, wildtype NCI-H1975, vector and on tumors treated with constructs desARE3’UTR ERBB2-1, 3 and 30. T-test of WT vs desARE3’UTRERBB2-1,3,30 (p=0.023, 0.019, 0.0145). T-test of Vector vs desARE3’UTR ERBB2-1,3,30 (p=0.0242, 0.0112, 0.0148)
7. Immunofluorescence pictures show XRN1 expression (stained in red) and nuclei (DAPI-blue) on xenograft tumors, wildtype NCI-H1975, vector and on tumors treated with constructs desARE3’UTR ERBB2-1, 3 and 30. Magnification (20x).
8. Bar chart show quantification of UPF3B protein fluorescence intensity on xenograft tumors, wildtype NCI-H1975, vector and on tumors treated with constructs desARE3’UTR ERBB2-1, 3 and 30. T-test of WT vs desARE3’UTRERBB2-1, 3, 30 (p=0.027, 0.0475, 0.0054). T-test on Vector vs desARE3’UTR ERBB2-1,3,30 (p=0.028, 0.0489, 0.0055)

### Table 1. Comparison of ShRNAi, CRISPR and mRNA 3’UTR destabilization in targeting HER2 in colorectal carcinoma HCT116.

| **Transcript perturbation method** | **Publication** | **Target** | **Cell line** | **Outcome** | **Associated kinases outcome** |
| --- | --- | --- | --- | --- | --- |
| Genomewide ShRNA | Vizeacoumar F et al Mol Syst Biol 2013 | EGFR, ERBB2 | HCT116 | ERBB2 not downregulated | YES1 not downregulated |
| Genome wide CRISPR KO | Hart T et al Cell 2015 | EGFR , ERBB2 | HCT116 | EGFR and ERBB2 not downregulated |  |
| Genome wide CRISPR KO plus shRNA | Martin T et al, Cell Rep 2017 | EGFR , ERBB2 | HCT116 | EGFR and ERBB2 not downregulated | YES1 not downregulated |
| desARE 3'UTR ERBB2-1,3 | Awah CU et al 2022, This Publication | EGFR , ERBB2 | HCT116 | ERBB2 downregulated | YES1 downregulated |
